# RCF1-dependent respiratory supercomplexes are integral for lifespan-maintenance in a fungal ageing model

**DOI:** 10.1038/srep12697

**Published:** 2015-07-29

**Authors:** Fabian Fischer, Christodoulos Filippis, Heinz D. Osiewacz

**Affiliations:** 1 Johann Wolfgang Goethe University, Faculty for Biosciences & Cluster of Excellence ‘Macromolecular Complexes’ Frankfurt, Institute of Molecular Biosciences, Max-von-Laue-Str. 9, 60438 Frankfurt, Germany

## Abstract

Mitochondrial respiratory supercomplexes (mtRSCs) are stoichiometric assemblies of electron transport chain (ETC) complexes in the inner mitochondrial membrane. They are hypothesized to regulate electron flow, the generation of reactive oxygen species (ROS) and to stabilize ETC complexes. Using the fungal ageing model *Podospora anserina*, we investigated the impact of homologues of the *Saccharomyces cerevisiae* respiratory supercomplex factors 1 and 2 (termed PaRCF1 and PaRCF2) on mtRSC formation, fitness and lifespan. Whereas PaRCF2’s role seems negligible, ablation of PaRCF1 alters size of monomeric complex IV, reduces the abundance of complex IV-containing supercomplexes, negatively affects vital functions and shortens lifespan. *PaRcf1* overexpression slightly prolongs lifespan, though without appreciably influencing ETC organization. Overall, our results identify PaRCF1 as necessary yet not sufficient for mtRSC formation and demonstrate that PaRCF1-dependent stability of complex IV and associated supercomplexes is highly relevant for maintenance of the healthy lifespan in a eukaryotic model organism.

Convincing evidence exists that mitochondrial dysfunction plays a key role in biological ageing and various age-related human pathologies^1^,^2^,^3^,^4^,^5^. It has long been suggested that the root cause for the progressive decline of mitochondrial function is the age-dependent accumulation of reactive oxygen species, which inevitably arise during oxidative phosphorylation^6^. The primary ROS superoxide anion (O_2_^.−^) is produced at complexes I and III of the ETC and can give rise to the secondary ROS hydrogen peroxide which in turn can lead to the formation of the highly reactive hydroxyl radical through the Fenton reaction. Both O_2_^.−^ and the hydroxyl radical are able to damage proteins, lipids and DNA. Consequently, all ROS are harmful to mitochondria when present in excess^7^. Recently, this narrow view of ROS solely as damaging agents has been challenged by counter-intuitive and contradictory experimental observations^8^,^9^. These may, at least in part, result from the fact that low levels of ROS are essential for cellular signalling and to control developmental processes^10^,^11^. A balanced generation and degradation of ROS is therefore highly important to keep biological systems functional over time. In this context, maintaining integrity of the ETC is crucial^5^.

In their seminal paper published in 2000, Schägger and Pfeiffer convincingly demonstrated the existence of stoichiometric assemblies of individual ETC complexes, so called mitochondrial respiratory supercomplexes or respirasomes, in yeast and mammalian mitochondria^12^. The ‘plasticity model’ of ETC organization is based on additional observations of considerable variations in mtRSC species and hypothesizes, that individual ETC complexes and supercomplexes can exist side by side in the inner mitochondrial membrane^13^,^14^. This view is supported by a growing number of studies demonstrating mitochondrial supercomplexes to be functional units of respiration assumed to be important for facilitating and directing electron flow by substrate channeling and consequently, to regulate the production of ETC-derived ROS. Additionally, there is evidence for a reciprocal dependence of supercomplex formation and the assembly and stabilization of individual ETC complexes, most importantly complex I^14^,^15^,^16^,^17^.

The proteins RCF1 and RCF2, members of the ‘hypoxia-inducible gene 1’ (HIG1) protein family, were recently identified in *S. cerevisiae* as being important constituents of mtRSCs^18^,^19^,^20^. ScRCF1 in particular was revealed as a crucial component for stabilization of the III_2_IV_2_ supercomplex^18^,^19^,^20^,^21^, while ScRCF2 appears to have a less prominent function^19^,^20^. The Rutter group also demonstrated a loss of complex IV-containing supercomplexes in mitochondria extracted from C2C12 mouse myoblast cells after knockdown of the mammalian RCF1 homologue HIG2A^18^.

Despite these notable insights, the *in vivo* relevance of mitochondrial supercomplexes remains to be clarified in detail. A destabilization of mtRSCs has already been linked to the development of at least one complex human disorder known as Barth syndrome^22^,^23^. It seems likely, owing to the central role of mitochondria in health and disease^5^, that similar connections will be unravelled in the near future^16^. To this end, it is an important task to determine the relationship between altered supercomplexes, mitochondrial function and impact on the organism as a whole.

The filamentous ascomycete *P*. *anserina* is characterized by a limited lifespan, a clear mitochondrial aetiology of ageing and has been extensively analysed as a simple model for organismal ageing^24^. Its ETC features, in contrast to that of *S*. *cerevisiae*, both complex I as well as an alternative terminal oxidase (AOX) that can be activated to bypass complexes III and IV by directly transferring electrons from ubiquinol to oxygen^25^. In the wild type, three major supercomplexes (I_1_III_2_IV_0–2_) can be resolved by blue native polyacrylamide gel electrophoresis (BN-PAGE)^26^. Genetic disruptions of complexes III and/or IV in *P*. *anserina* have been shown to result in absence of the corresponding supercomplexes^26^,^27^ and enhanced AOX-dependent respiration, with mutant strains consistently displaying increased lifespans^25^,^27^,^28^,^29^.

Here we describe the role of RCF1 and RCF2 in *P*. *anserina*. Deletion of *PaRcf2*, encoding the RCF2 homologue, has no pronounced effect on the phenotype, while ablation of PaRCF1 shifts the complex IV monomer almost exclusively to a form that migrates faster during BN-PAGE than that of the wild type. In addition, the abundance of I_1_III_2_IV_1–2_ supercomplexes is strongly reduced. Concomitant with these changes, mitochondrial integrity as well as vital characteristics of the *PaRcf1* deletion strain, such as growth rate and fertility, are impaired and lifespan, despite activation of the alternative respiratory pathway, is shortened. Overall, our results identify RCF1-stabilized complex IV and complex IV-containing supercomplexes as crucial ETC components in *P*. *anserina*, establishing a novel connection between their destabilization, impaired mitochondrial function and a negative impact on health and lifespan.

## Results

### Deletion of *PaRcf1* disturbs complex IV and supercomplexes

**Figure 1:**
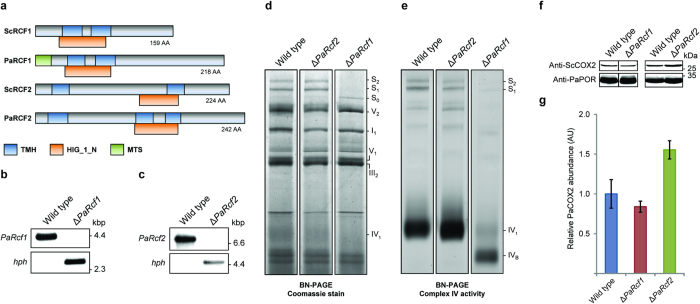
Deletion of *PaRcf1* alters ETC composition. (**a**) Comparison of *S*. *cerevisiae* and *P*. *anserina* RCF1 and RCF2 homologues. All proteins contain a hypoxia induced protein conserved region (HIG_1_N) domain and two or three predicted transmembrane helices (TMH). PaRCF1 additionally has a predicted mitochondrial targeting sequence (MTS). (**b**) Southern blot analysis of HindIII-digested genomic DNA (gDNA) from wild type and Δ*PaRcf1*. A *PaRcf1*-specific hybridization probe detects the 4217 bp *PaRcf1*-fragment only in wild-type gDNA. A 2659 bp fragment containing the hygromycin B phosphotransferase (*hph*) gene is detected only in gDNA of Δ*PaRcf1*. (**c**) Southern blot verification of Δ*PaRcf2*. The *PaRcf2*-fragment is 7153 bp and the *hph*-fragment 4667 bp in size. (**d**) Representative BN-PAGE analysis of mitochondrial protein extracts from the indicated strains. The I_1_III_2_IV_0–2_ (S_0–2_) supercomplexes, dimeric complexes III and V (III_2_ and V_2_) as well as monomeric complexes I, IV and V were visualized by Coomassie staining. (**e**) Representative complex IV ‘in-gel’ activity assay with mitochondrial protein extracts from the indicated strains. (**f**) Representative western blot analysis of mitochondrial protein extracts from wild type, Δ*PaRcf1* and Δ*PaRcf2*. A ScCOX2-specific antibody was used to detect the ~29 kDa PaCOX2 subunit of complex IV. PaPORIN (PaPOR) was detected as a loading control. (**g**) Quantitative western blot analysis of mitochondrial protein extracts from wild type (*n* = 4), Δ*PaRcf1* (*n* = 4) and Δ*PaRcf2* (*n* = 4). The PaCOX2 abundance was normalized to that of PaPOR and the mean wild-type abundance was defined as 1. Data given in parentheses are mean PaCOX2 abundance ± s.e.m. in arbitrary units (AU).

The genome of *P*. *anserina* is fully sequenced (http://podospora.igmors.u-psud.fr). Using BLAST searches (http://podospora.igmors.u-psud.fr/blast.php), we identified a homologue of ScRCF1 (UniProt: Q03713), murine HIG2A (UniProt: Q9CQJ1) and human HIG2A (UniProt: Q9BW72) that we termed PaRCF1 (*P*. *anserina* accession number: Pa_5_5310; UniProt: Q875C2) as well as a homologue of ScRCF2 (UniProt: P53721) termed PaRCF2 (*P*. *anserina* accession number: Pa_1_4450; UniProt: B2AAL7). PaRCF1 and PaRCF2 share an amino acid (AA) sequence identity of 26% and 27% and a similarity of 40% and 44% compared with the respective homologues of *S*. *cerevisiae* (Supplementary Fig. S1). PaRCF1 has a length of 218 AA with a predicted 18 AA N-terminal mitochondrial targeting sequence (MTS) and a calculated mass of ~22 kDa for the mature protein. Similar to ScRCF1 it contains a ‘hypoxia induced protein conserved region’ domain (HIG_1_N) and two predicted transmembrane helices (TMH). PaRCF2 has a length of 242 AA, a calculated mass of ~27 kDa and contains a HIG_1_N domain and three predicted TMHs (Fig. 1a and Supplementary Fig. S1).

*PaRcf1* and *PaRcf2* deletion strains (Δ*PaRcf1* and Δ*PaRcf2*) were generated according to the method described by El-Khoury and colleagues, with the respective wild-type gene’s ORF replaced by a hygromycin B resistance gene^30^. The deletion strains were verified by Southern blot analysis (Fig. 1b,c and Supplementary Fig. S2).

To analyse the importance of PaRCF1 and PaRCF2 for maintaining integrity of the ETC, mitochondrial protein extracts from wild type (*n* = 4), Δ*PaRcf1* (*n* = 3) and Δ*PaRcf2* (*n* = 4) were compared using BN-PAGE (Fig. 1d, Table 1 and Supplementary Fig. S3). Deletion of *PaRcf2* had no discernible effect on overall ETC composition. *PaRcf1* deletion, however, led to marked changes. Specifically, relative abundance of the I_1_III_2_IV_2_ (S_2_) supercomplex (0.07 ± 0.01 AU; *P* = 0.02 by two-tailed Student’s *t*-test) and of the I_1_III_2_IV_1_ (S_1_) supercomplex (0.03 ± 0.02 AU; *P* = 6.8E-04 by two-tailed Student’s *t*-test) in Δ*PaRcf1* was significantly reduced, while relative abundance of the I_1_III_2_IV_0_ (S_0_) supercomplex (15.30 ± 2.58 AU; *P* = 0.03 by two-tailed Student’s *t*-test) was significantly increased. Free complex IV monomer was undetectable in mitochondrial protein extracts from Δ*PaRcf1*, though it should be noted that even in wild type and Δ*PaRcf2* monomeric complex IV is detectable only by a diffuse and rather faint band after BN-PAGE and Coomassie staining. A complex IV ‘in-gel’ activity assay (Fig. 1e, Table 2 and Supplementary Fig. S4) was used to measure relative distribution of complex IV activity within each individual strain and confirmed the near total absence of the S_2_ and S_1_ supercomplexes in Δ*PaRcf1*. It also revealed that monomeric complex IV in Δ*PaRcf1* is almost exclusively present in a faster migrating form (IV_B_) than in the wild type or in Δ*PaRcf2*.

Relative abundance of PaCOX2, an essential complex IV subunit encoded by the mitochondrial genome, was found to be slightly reduced in mitochondrial protein extracts from Δ*PaRcf1* (0.84 ± 0.07 AU; *P* = 0.45 by two-tailed Student’s *t*-test), while in contrast being increased in those from Δ*PaRcf2* (1.55 ± 0.11 AU; *P* = 4.7E-02 by two-tailed Student’s *t*-test) compared to the wild type (1.00 ± 0.18 AU; Fig. 1f,g and Supplementary Fig. S5).

### ETC alterations in Δ*PaRcf1* impair mitochondrial integrity

**Figure 2:**
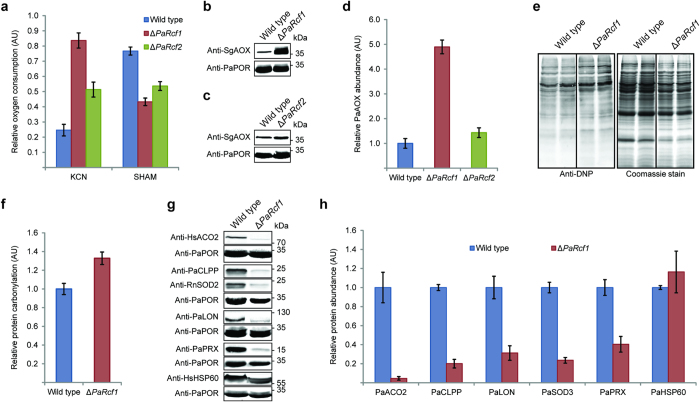
Mitochondrial function is impaired in absence of PaRCF1. (**a**) Relative complex IV- and AOX-dependent oxygen consumption of wild-type (*n* = 3), Δ*PaRcf1* (*n* = 3) and Δ*PaRcf2* (*n* = 3) mycelium after treatment with a complex IV (KCN) or AOX (SHAM) inhibitor. Total oxygen consumption of the respective untreated strain’s mycelium was defined as 1. Data are mean oxygen consumption ± s.e.m in arbitrary units (AU). (**b**) Representative western blot analysis of mitochondrial protein extracts from wild type and Δ*PaRcf1*. A SgAOX-specific antibody was used to detect the ~34 kDa PaAOX protein. PaPORIN (PaPOR) was detected as a loading control. (**c**) Representative western blot analysis of mitochondrial protein extracts from wild type and Δ*PaRcf2*. (**d**) Quantitative western blot analysis of mitochondrial protein extracts from wild type (*n* = 4), Δ*PaRcf1* (*n* = 4) and Δ*PaRcf2* (*n* = 4). The PaAOX abundance was normalized to that of PaPOR and the mean wild-type abundance was defined as 1. Data are mean PaAOX abundance ± s.e.m. in AU. (**e**) Representative western blot analysis of mitochondrial protein extracts from wild type and Δ*PaRcf1* treated with the ‘OxyBlot™ Protein Oxydation Detection Kit’ (Merck Millipore). Carbonyl groups of oxidized proteins derivatized to 2,4-dinitrophenylhydrazone (DNP-hydrazone) were detected with a DNP-specific antibody. The Coomassie stained gel after blotting served as a loading control. (**f**) Quantitative western blot analysis of mitochondrial protein extracts from wild type (*n* = 6) and Δ*PaRcf1* (*n* = 6). The mean wild-type protein carbonylation was defined as 1. Data are mean protein carbonylation ± s.e.m. in AU. (**g**) Representative western blot analyses of mitochondrial protein extracts from wild type and Δ*PaRcf1* using the indicated antibodies. PaACO2 and PaHSP60 were detected with antibodies directed against the human homologues (Anti-HsACO2 or Anti-HsHSP60). PaSOD3 was detected with a rat SOD2 antibody (Anti-RnSOD2). PaPOR was detected as a loading control. (**h**) Quantitative western blot analyses of mitochondrial protein extracts from wild type (*n* = 4) and Δ*PaRcf1* (*n* = 4). Protein abundances were normalized to that of PaPOR and the mean wild-type abundances were defined as 1. Data are mean protein abundance ± s.e.m. in AU.

To assess the functional consequences of *PaRcf1* and *PaRcf2* deletion for mitochondrial respiration, we measured oxygen consumption of wild type (*n* = 3), Δ*PaRcf1* (*n* = 3) and Δ*PaRcf2* (*n* = 3) mycelium with and without the addition of the specific respiratory inhibitors KCN, inhibiting complex IV, and salicylhydroxamic acid (SHAM), inhibiting AOX (Fig. 2a and Supplementary Fig. S6). Notably, total absolute oxygen consumption of Δ*PaRcf1* was tendentially increased compared to the wild type (Supplementary Fig. S6). Similar effects have previously been observed in other *P*. *anserina* ETC mutants, likely reflecting inefficient oxygen utilization for ATP generation^31^. Sequential addition of both inhibitors in either order (i.e. first KCN and then SHAM or first SHAM and then KCN) almost completely inhibited oxygen consumption in all strains. Therefore, the majority of oxygen in *P*. *anserina* is consumed during mitochondrial respiration (Supplementary Fig. S6). Relative oxygen consumption after inhibition of complex IV with KCN was significantly higher in Δ*PaRcf1* (0.83 ± 0.07 AU; *P* = 5.2E-03 by two-tailed Student’s *t*-test) than in wild type (0.25 ± 0.08 AU), i.e. Δ*PaRcf1* exhibits, in accord with the results obtained by BN-PAGE (Fig. 1d,e and Tables 1 and 2), a decrease in complex IV-dependent respiration. In contrast, inhibition of AOX with SHAM resulted in significantly lower relative oxygen consumption in Δ*PaRcf1* (0.43 ± 0.03 AU; *P* = 7.0E-03 by two-tailed Student’s *t*-test) than in wild type (0.76 ± 0.05 AU). Interestingly, the effect of complex IV inhibition by KCN in Δ*PaRcf1* was more pronounced if AOX was first inhibited by SHAM and vice versa (Supplementary Fig. S6). This probably reflects an adaptive upregulation of either respiratory pathway after inhibition of the other pathway during oxygen consumption measurements.

In conclusion, these observations indicate an upregulation of the alternative respiratory pathway in Δ*PaRcf1*. Similar yet less pronounced changes in absolute and relative oxygen consumption after addition of KCN or SHAM were also observed in Δ*PaRcf2* (Fig. 2a and Supplementary Fig. S6), despite the lack of visible alterations in ETC composition (Fig. 1d,e and Tables 1 and 2).

Comparison of PaAOX abundance in mitochondrial protein extracts from wild type (*n* = 4), Δ*PaRcf1* (*n* = 4) and Δ*PaRcf2* (*n* = 4) by western blot analysis (Fig. 2b–d and Supplementary Fig. S7) revealed significant changes only in Δ*PaRcf1*, where relative PaAOX abundance was considerably increased (4.89 ± 0.28 AU; *P* = 5.5E-03 by two-tailed Student’s *t*-test). As no obvious deviations in Δ*PaRcf2*’s phenotype from that of the wild type, apart from the changes in oxygen consumption (Fig. 2a), were identified (see also below), we subsequently focused on Δ*PaRcf1*.

Different *P*. *anserina* ETC mutants display decreased mitochondrial ROS and an increased lifespan concomitant with enhanced activation of the alternative respiratory pathway^25^,^28^. Consequently, the observation that respiration in Δ*PaRcf1* is primarily AOX-dependent suggested that superoxide mediated damage to proteins might be reduced in this strain. Contrary to this assumption we found that Δ*PaRcf1* had a slight but nonetheless significant increase in mitochondrial protein carbonylation (1.33 ± 0.07 AU; *n* = 6; *P* = 4.6E-03 by two-tailed Student’s *t*-test), possibly reflecting impairments in ROS scavenging mechanisms and/or in the clearance of damaged mitochondrial proteins (Fig. 2e,f).

Western blot analyses comparing relative protein abundances in mitochondrial protein extracts from wild type (*n* = 4) and Δ*PaRcf1* (*n* = 4) indeed revealed significant reductions of mitochondrial matrix proteases PaCLPP (0.20 ± 0.05 AU; P = 1.6E-05 by two-tailed Student’s *t*-test) and PaLON (0.31 ± 0.08 AU; *P* = 4.2E-03 by two-tailed Student’s *t*-test) as well as of mitochondrial superoxide dismutase PaSOD3 (0.24 ± 0.03 AU; *P* = 1.2E-04 by two-tailed Student’s *t*-test) and peroxiredoxin PaPRX (0.40 ± 0.08 AU; *P* = 2.3E-03 by two-tailed Student’s *t*-test) in Δ*PaRcf1*, while level of the chaperone PaHSP60 (1.16 ± 0.22 AU) remained unchanged (Fig. 2g,h and Supplementary Fig. S8). In addition, relative abundance of mitochondrial aconitase PaACO2 was also found to be strongly and significantly reduced (0.05 ± 0.02 AU; *P* = 9.0E-03 by two-tailed Student’s *t*-test) in the *PaRcf1* deletion strain (Fig. 2g,h and Supplementary Fig. S8).

Surprisingly, despite the strong reduction of PaSOD3 protein abundance in Δ*PaRcf1* by almost 80% (Fig. 2g,h), visualization of PaSOD3-activity in mitochondrial protein extracts from *P*. *anserina* wild type and Δ*PaRcf1* with an SOD ‘in-gel’ activity assay revealed no observable differences between the two strains (Supplementary Fig. S9). This result suggests that the remaining PaSOD3 in Δ*PaRcf1* is highly active to counteract oxidative stress.

### Δ*PaRcf1* is unable to maintain a healthy lifespan

To date, consequences for organismal health following a targeted genetic disruption of mitochondrial respiratory supercomplexes are scarcely studied. *P*. *anserina* RCF1, as demonstrated in this study, is critically involved in stabilizing complex IV. Concomitant with a destabilization of complex IV in Δ*PaRcf1*, abundance of associated supercomplexes is also strongly reduced (Fig. 1d,e and Tables 1 and 2). Presumably as a consequence thereof, mitochondrial integrity in the *PaRcf1* deletion strain is negatively affected (Fig. 2a–h). To better understand the resulting impact on the organism as a whole, several vital characteristics of Δ*PaRcf1* were assessed.

**Figure 3:**
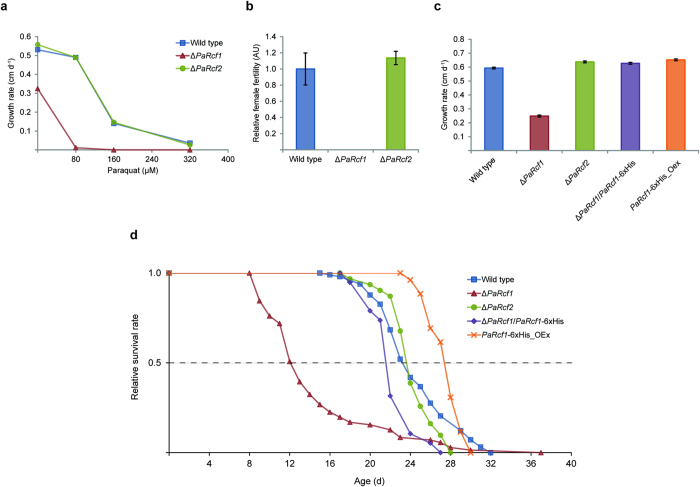
Vital characteristics and lifespan-maintenance are heavily impaired in Δ*PaRcf1*. (**a**) Growth rate of wild type (*n* = 20), Δ*PaRcf1* (*n* = 20) and Δ*PaRcf2* (*n* = 20) on M2-medium with 0, 80, 160 or 320 μM of paraquat. Data are mean growth rate in centimetres per day. (**b**) Female fertility of wild type (*n* = 4), Δ*PaRcf1* (*n* = 4) and Δ*PaRcf2* (*n* = 4). The mean wild-type female fertility was defined as 1. Data are mean female fertility ± s.e.m. in AU. (**c**) Growth rate of wild type (*n* = 98), Δ*PaRcf1* (*n* = 71), Δ*PaRcf2* (*n* = 31), Δ*PaRcf1*/*PaRcf1*-6xHis (*n* = 19) and *PaRcf1*-6xHis_OEx (*n* = 26). Data are mean growth rate ± s.e.m. in centimetres per day. (**d**) Lifespan of wild type (*n* = 98), Δ*PaRcf1* (*n* = 71), Δ*PaRcf2* (*n* = 31), Δ*PaRcf1*/*PaRcf1*-6xHis (*n* = 19) and *PaRcf1*-6xHis_OEx (*n* = 26).

It is reasonable to assume that Δ*PaRcf1*, showing increased oxidative protein damage (Fig. 2e,f) and a reduction of key enzymes involved in ROS scavenging (Fig. 2g,h), should be more susceptible to oxidative stressors. This was indeed true for paraquat, known to generate O_2_^.−^ at the ETC^32^, which almost completely inhibited growth of the *PaRcf1* deletion strain already at a concentration of 80 μM. At this concentration, growth rate of wild type and Δ*PaRcf2* remained nearly unaffected (Fig. 3a). Other stressors, namely H_2_O_2_ and CuSO_4_, had no intensified effect on Δ*PaRcf1* (Supplementary Fig. S10). Next, influence of 20 μM paraquat on survival of the *PaRcf1* and *PaRcf2* deletion strains was investigated. Previous work of our laboratory showed that, similar to what has been observed in *Caenorhabditis elegans*^33^,^34^, low doses of paraquat can considerably prolong lifespan of the *P*. *anserina* wild-type strain^35^. Interestingly, this effect was completely abrogated in Δ*PaRcf1*, while still being preserved in Δ*PaRcf2* (Supplementary Fig. S11).

As the mammalian RCF1 homologue HIG1A is known to be transcriptionally regulated^36^, we speculated that *PaRcf1* expression might be induced by low doses of paraquat and that PaRCF1 is possibly involved in mediating paraquat-triggered lifespan extension. Relative *PaRcf1* expression in the wild type was, however, not elevated after cultivation with 20 μM paraquat (Supplementary Fig. S11).

The *PaRcf1* deletion strain was further characterized by female infertility (Fig. 3b) and a significant reduction of its growth rate on standard medium by 58% (0.25 ± 0.01 cm d^−1^; *n* = 69; *P* = 2.2E-27 by two-tailed Wilcoxon rank-sum test; Fig. 3c and Table 3). Perhaps the most remarkable characteristic of Δ*PaRcf1*’s phenotype was a significant 40% shortening of its mean lifespan (14.5 ± 0.7 d; *n* = 71; *P* = 3.4E-19 by two-tailed Wilcoxon rank-sum test; Fig. 3d and Table 3). Deletion of *PaRcf2*, aside from a slightly elevated growth rate, again led to no distinct deviations from the wild type under any of the conditions tested (Fig. 3a–d and Table 3).

To determine the specificity of the observed effects, we complemented Δ*PaRcf1* by introducing a C-terminally 6xHis-tagged variant of *PaRcf1* under control of the native promotor and terminator. Presence of the recombinant gene in the complemented strain (Δ*PaRcf1*/*PaRcf1*-6xHis) was verified by Southern blot analysis (Supplementary Fig. S12). Relative expression of *PaRcf1*-6xHis was similar to that of *PaRcf1* in the wild type (Supplementary Fig. S12) and the recombinant protein could be detected in mitochondrial protein extracts from Δ*PaRcf1*/*PaRcf1*-6xHis (Supplementary Fig. S12). The complemented strain was again fertile and displayed a wild-type like growth rate (Fig. 3c and Table 3) and lifespan (Fig. 3d and Table 3).

Finally, since absence of PaRCF1 had such a dramatic negative effect on health and lifespan, we investigated whether overexpression of *PaRcf1* might be beneficial for *P*. *anserina*. A strain expressing *PaRcf1*-6xHis under control of a constitutive promotor in the wild-type background (*PaRcf1*-6xHis_OEx) was generated and verified by Southern and western blot analysis (Supplementary Fig. S13). Relative *PaRcf1* expression (20-fold; *P* = 3.6E-02 by two-tailed Student’s *t*-test) and relative PaRCF1-6xHis protein abundance (17-fold; *P* = 6.5E-17 by two-tailed Student’s *t*-test) were significantly elevated compared with the wild type or Δ*PaRcf1*/*PaRcf1*-6xHis, confirming stable overexpression of the construct (Supplementary Fig. S13). While paraquat resistance of *PaRcf1*-6xHis_OEx remained essentially unchanged (Supplementary Fig. S13), both its growth rate (0.65 ± 0.01 cm d^−1^; *n* = 26; *P* = 8.3E-06 by two-tailed Wilcoxon rank-sum test) and lifespan (27.6 ± 0.3 d; *n* = 26; *P* = 1.1E-02 by two-tailed Wilcoxon rank-sum test) were slightly but significantly increased by +10% and +13%, respectively (Fig. 3c,d and Table 3). These changes were, however, not correlated with a discernible increase in supercomplexes as assayed by BN-PAGE, thereby suggesting that PaRCF1 alone is not sufficient to markedly elevate formation of mtRSCs (Supplementary Fig. S13 and Supplementary Table S1).

## Discussion

The mitochondrial protein RCF1 has recently been identified in three independent studies as a novel stabilizing component of the III_2_IV_2_ supercomplex in *S*. *cerevisiae*. It was further demonstrated to preferentially interact with cytochrome c oxidase and to be important for maintaining activity of this complex. In addition, ScRCF1 is also able to independently associate with complex III^18^,^19^,^20^,^21^. Despite these insights, the detailed nature and function of ScRCF1 are not entirely clear and the authors’ interpretations are somewhat conflicting. Based on the observation that ScRCF1 maintains association with complex III subunits even in absence of assembled complex IV, one study concluded that ScRCF1 is a true supercomplex assembly factor and not a subunit of complex IV^18^. In contrast, Vukotic and colleagues described ScRCF1 as more likely being a new subunit of at least one particular complex IV isoform in which it mediates contact with complex III^20^. In yet another study, ScRCF1 was recognized as a possible cytochrome c oxidase assembly and regulatory factor but it was also noted that the ability of ScRCF1 to reliably interact with complex III is unique for a potential complex IV component^19^.

In our study, we show that RCF1 in *P*. *anserina* is absolutely crucial for the stability of complex IV and that PaRCF1 ablation leads to a strong reduction of supercomplex abundance. While it is not yet clear whether PaRCF1 directly interacts with supercomplexes to induce their formation, its impact on overall ETC organization is even more pronounced than what has been observed for RCF1 in *S*. *cerevisiae*^18^,^19^,^20^ or the mammalian RCF1 homolog HIG2A in mice^18^. In contrast, deletion of the gene coding for the *P*. *anserina* RCF2 homologue led to no pronounced phenotype and did not measurably affect complex IV integrity or ETC organization. While intermediate activation of the alternative respiratory pathway in Δ*PaRcf2* clearly argues for a role of PaRCF2 in maintaining standard respiration, it appears questionable whether PaRCF2 is indeed a functional homologue of ScRCF2, which was demonstrated to have partially overlapping functions with ScRCF1^19^,^20^. Variations regarding the relative importance of RCF1 or RCF2 in different model organisms are likely explained by varying ETC compositions, with the most obvious deviation being the complete lack of complex I in *S*. *cerevisiae*^37^,^38^. In mammals, which do not possess a RCF2 homologue, complex I has been described as being stabilized by supercomplex formation with complexes III and IV and at the same time to provide a scaffold for efficient respirasome assembly^39^. There is convincing evidence that in *P*. *anserina* the stability of complex I is not dependent on complexes III and/or IV, possibly due to such specific features as presence of the AOX and complex I dimerization^27^. It can be speculated that complex III and IV interaction in the context of supercomplex formation in turn is less reliant on complex I and mainly dependent on the presence of other factors such as PaRCF1.

Probably the most striking change in the ETC of Δ*PaRcf1* is the appearance of a faster migrating complex IV variant (IV_B_) which is found almost exclusively instead of fully assembled complex IV. While a comparable phenomenon has so far not been observed in *S*. *cerevisiae*, Chen and colleagues report a similar yet less pronounced increase in incomplete complex IV after knockdown of mammalian HIG2A in C2C12 mouse myoblast cells^18^. The appearance of the IV_B_ variant seems to be highly specific for absence of PaRCF1, as it has not been detected nearly as prominently or at all in other *P*. *anserina* ETC mutants impaired in supercomplex formation^26^,^27^.

In light of these insights, it is logical to conclude that PaRCF1 is a subunit or a specific regulatory factor of a particular complex IV variant, whose assembly likely precedes and in fact appears to be a prerequisite for formation of the I_1_III_2_IV_1-2_ supercomplexes. Thus, decreased formation of supercomplexes in Δ*PaRcf1* could well be a direct consequence of incorrect complex IV assembly. To further the understanding of supercomplex assembly and regulation, it arises as an important future task to address the specialized role and heterogeneous nature of distinct ETC complex variants in different organisms, especially regarding their importance for higher order organization of the respiratory chain.

The majority of studies concerning mitochondrial respiratory supercomplexes have focused on their biochemical, structural and kinetic properties. Consequently, the contribution of mtRSCs to mitochondrial function and organismal integrity is as yet not well understood. One of the first hints at the relevance of mtRSCs *in vivo* has come from the insight that human Barth syndrome, a hereditary cardiomyopathy occurring exclusively in males, is linked to a destabilization of supercomplexes caused by abnormal mitochondrial cardiolipin^22^,^23^. In addition, a recent study in mice demonstrated the necessity of a supercomplex assembly factor for the regulation of energy metabolism in muscle^40^. To a lesser extent mtRSCs have also been implicated in more complex phenomena such as cancer progression, neurodegeneration and ageing, though causative evidence and understanding of the underlying molecular pathways is still largely missing^41^.

Our results clearly demonstrate that RCF1-dependent stability of complex IV and presence of associated supercomplexes in *P*. *anserina* is important for mitochondrial function and organismal integrity. On a physiological level, in good agreement with our biochemical observations, the *PaRcf1* deletion strain displays reduced complex IV-dependent respiration and activation of the alternative respiratory pathway. Other *P*. *anserina* mutants lacking I_1_III_2_IV_1–2_ supercomplexes and respiring primarily via the AOX, owing to defects in complex III and/or complex IV, very consistently display prolonged lifespans^25^,^27^,^28^,^29^. In striking contrast to these previous observations, not only are vital functions and superoxide resistance of the *PaRcf1* deletion strain negatively affected but its lifespan is also markedly reduced by nearly 50%. On a molecular level, this adverse overall impact is correlated with increased oxidative damage of mitochondrial proteins. This is a further distinguishing feature of Δ*PaRcf1*, as a switch to AOX-dependent respiration in *P*. *anserina* mutants was generally found to result in a decreased ROS burden^25^,^28^. A likely explanation for these findings, aside from an increase in ROS production itself, is the bold reduction of several ROS scavenging and protein quality control components in mitochondria of Δ*PaRcf1*. The marked reduction of PaSOD3 protein abundance in mitochondria of the *PaRcf1* deletion strain seems to be of particular significance because, quite unexpectedly, PaSOD3 activity in Δ*PaRcf1* still appears almost identical to that in the wild type. Together with the observation that Δ*PaRcf1* is barely able to survive any additional paraquat-induced oxidative stress, this strongly suggests that PaSOD3 in Δ*PaRcf1* is working at the limits of its capacity. Interestingly, deletion of *S*. *cerevisiae Rcf1* increased mitochondrial SOD abundance^18^. The inability of Δ*PaRcf1* to likewise upregulate its mitochondrial SOD in response to elevated endogenous oxidative stress underscores the dramatic effect on respiratory chain integrity and activity following ablation of PaRCF1 and likely reflects impairment of mitochondrial protein import and/or cytoplasmic protein synthesis due to reduced availability of ATP.

Though additional studies are necessary to address these phenomena in detail, it can already be inferred that they must be linked to conditions or properties only present in Δ*PaRcf1* and not in other *P*. *anserina* ETC mutants. The aforementioned unprecedented and near exclusive emergence of a faster migrating complex IV variant in absence of PaRCF1 meets this criterion. Of note, Vukotic and colleagues proposed that a specific complex IV variant, absent in mitochondria of the *S*. *cerevisiae Rcf1* deletion strain, serves to protect the ETC from excess ROS generation and that lack of ScRCF1 might thus lead to malfunction and ROS production in a catalytic manner^20^. Despite several crucial differences in the ETC of *S*. *cerevisiae* and *P*. *anserina*, our results are readily compatible with this model. Beyond that, we identified a novel connection between destabilization of supercomplexes, impaired mitochondrial function and adverse effects on health and lifespan in a simple eukaryotic model organism. As the overall respiratory chain composition of *P*. *anserina* is comparable to that of mammals, it will be of great interest to investigate whether similar relationships exist in them as well.

## Methods

### *P*. *anserina* strains and cultivation

In the present study, the *P. anserina* wild-type strain ‘s’^42^ and newly generated *PaRcf1* and *PaRcf2* deletion strains (Δ*PaRcf1* and Δ*PaRcf2*) as well as a complemented *PaRcf1* deletion strain (Δ*PaRcf1*/*PaRcf1*-6xHis) and a strain overexpressing *PaRcf1*-6xHis in the wild-type background (*PaRcf1*-6xHis_OEx) were used. All mutant strains are in the genetic background of the wild-type strain ‘s’. Strains were grown on standard cornmeal agar (BMM) at 27 °C under constant light^43^.

### Cloning procedures and generation of *P. anserina* mutants

Deletion of *PaRcf1* and *PaRcf2* was performed with the method developed by El-Khoury and colleagues using the plasmid pKO7^30^,^44^. Briefly, approximately 1 kbp long fragments corresponding to the 5′ and 3′ regions of the respective gene of interest were amplified by PCR using sequence specific oligonucleotides with appropriate restriction site overhangs (*PaRcf1* 5′ region with NotI-PaRcf1_for3: 5′-ATGCGGCCGCCAATAGTGGCTGGGATTT-3′ and BcuI-PaRcf1_rev4: 5′-GCGCACTAGTCTGATCCAGGGAAGATCG-3′; *PaRcf1* 3′ region with ClaI-PaRcf1_for5: 5′-CGATCGATAAGTGGATGGCTGTAACG-3′ and XhoI-PaRcf1_rev6: 5′-ATCTCGAGCGCAAGCTCCGATTACTG-3′; *PaRcf2* 5′ region with BcuI-PaRcf2_for3: 5′-GCACTAGTTGGACCAGTCCTGTTGAG-3′ and PstI-PaRcf2_rev4: 5′-ATCTGCAGTGGGTGGAGACTGAAGAG-3′; *PaRcf2* 3′ region with ClaI-PaRcf2_for5: 5′-GCATCGATTCACGACACGATCTATCC-3′ and XhoI-PaRcf2_rev6: 5′-ATCTCGAGGCTGCTGAATCTGGTGAC-3′; restriction sites underlined) and then cloned into the plasmid pKO7 to flank a hygromycin B resistance gene. The resulting gene specific deletion vectors were used to transform *P*. *anserina* spheroblasts of the phleomycin resistant Δ*PaKu70* strain. Transformants were then selected by their hygromycin B resistance and crossed with the wild type to reintroduce the *PaKu70* gene. Offspring of these crosses were selected by their hygromycin B resistance and phleomycin sensitivity and further verified by Southern blot analysis.

For complementation of the *PaRcf1* deletion strain with C-terminally 6xHis-tagged PaRCF1, Δ*PaRcf1* spheroblasts were transformed with the plasmid pKO6-PaRcf1-6xHis containing a phleomycin resistance gene in the pKO6 vector backbone^45^ and the full-length *PaRcf1* gene under control of its native promotor and terminator, with a 6xHis-tag coding sequence added before the gene’s stop codon. Transformants were selected for phleomycin resistance and verified by Southern blot analysis. Strains with a single integration of pKO6-PaRcf1-6xHis were termed Δ*PaRcf1*/*PaRcf1*-6xHis. To construct the vector, the *PaRcf1* promotor (~1 kbp of the gene’s upstream region), gene and terminator (~500 bp of the gene’s downstream region) were amplified from genomic wild type DNA by PCR using the oligonucleotides EcoRI-PaRcf1_A (5′-TAGAATTCCGTGTCCGCCCATTCTCG-3′) and SpeI-PaRcf1_B (5′-ATACTAGTCCCACCACCGCAACCTAC-3′), introducing EcoRI and SpeI restriction sites (underlined). The amplicon was cloned into the pKO6 backbone (EcoRI/SpeI digested) to obtain the plasmid pKO6-PaRcf1. Using pKO6-PaRcf1 as a template, a 5′ fragment containing a portion of the *PaRcf1* promotor including a PstI restriction site, the full length gene sequence and part of the 6xHis-tag coding sequence was amplified by PCR using the oligonucleotides PaRcf1_Hisfor1 (5′-GTCGTTGAAGTCGCAAGAAG-3′) and PaRcf1_Hisrev2 (5′-GGTGGTGATGGTTCTTCGGGTCTTCTG-3′). A 3′ fragment containing the remaining 6xHis-tag coding sequence and the *PaRcf1* terminator including a BcuI restriction site was amplified by PCR using the oligonucleotides PaRCF1_Hisfor3 (5′-ATCACCATTAAGGGTGAAGTGGATGGC-3′) and PaRcf1_Hisrev4 (5′-GCCGCTCTAGAACTAGTCC-3′). The fragments were then cloned into the pKO6-PaRcf1 backbone (PstI/BcuI digested) to obtain pKO6-PaRcf1-6xHis.

To generate a *PaRcf1*-6xHis overexpressing strain, wild-type spheroblasts were transformed with the newly constructed plasmid pExMtterhph-PaRcf1-6xHis-OEx containing a hygromycin B resistance gene in the pExMtterhph vector backbone^46^ and the full-length *PaRcf1* gene under control of the strong constitutive metallothionein promoter and the metallothionein terminator, with a 6xHis-tag coding sequence added before the gene’s stop codon. Transformants were selected for hygromycin B resistance and verified by Southern blot analysis. Strains with a single integration of pExMtterhph-PaRcf1-6xHis-OEx were termed *PaRcf1*-6xHis_OEx. To construct the vector, the *PaRcf1* gene including the 6xHis-tag coding sequence was amplified by PCR using the oligonucleotides BglII-PaRcf1_for (5′-TAAGATCTATGTCGAACGGACCCCTCTC-3′) and XbaI-6xHis_rev (5′-GCTCTAGATTAATGGTGATGGTGGTGATG-3′) with pKO6-PaRcf1-6xHis as a template, introducing BglII and XbaI restriction sites (underlined). The amplicon was cloned into the pExMtterhph backbone (BamHI/XbaI digested) to obtain pExMtterhph-PaRcf1-6xHis-OEx.

### Transformation of *P. anserina* spheroblasts

The respective strain to be transformed was grown on BMM at 27 °C under constant light for 3 days and subsequently under the same conditions in liquid complete medium (CM) for 2 days (CM medium: 1 g/l KH_2_PO_4_, 0.5 g/l KCl, 0.5 g/l MgSO_4_ × 7 H_2_O, 10 g/l glucose, 3.7 g/l NH_4_Cl, 2 g/l tryptone, 2 g/l yeast extract and 1 g/l ZnSO_4_, FeCl_2_ and MnCl_2_; pH 6.5). 20 g of the resulting mycelium was washed with TPS buffer (5 mM Na_2_HPO_4_, 45 mM KH_2_PO_4_, 0.8 M sucrose; pH 5.5) and TPS buffer containing 20 mg/ml ‘Glucanex’ (Novozymes) was added to a final volume of 100 ml. After chopping the mixture in a ‘Waring Blendor’ the resulting suspension was incubated for 1.5 h at 35 °C. Following filtration through gauze and glass wool, the suspension was centrifuged for 10 min at 4.000 rpm to pelletise the spheroplasts and the pellet was washed three times with TPS buffer.

To regenerate spheroplasts, the pellet was recovered in TPS buffer and the spheroplasts were plated on regeneration agar (3.7 g/l NH_4_Cl, 2 g/l tryptone, 1 g/l casamino acids, 1 g/l yeast extract, 10 g/l glucose, 342.3 g/l sucrose, 1.5 g/l KH_2_PO_4_, 0.5 g/l KCl, 0.54 g/l MgSO_4_ and 1 mg/l MnSO_4_ × 1 H_2_O, FeSO_4_ × 7 H_2_O, CuSO_4_ × 5 H_2_O and ZnSO_4_ × 7 H_2_O) containing 100 μg/ml hygromycin B. After 7–10 days of growth, mycelia of developing cultures were transferred to BMM agar plates.

Integrative transformation of *P. anserina* spheroplasts was performed as described previously^47^.

### Lifespan determination

To determine the lifespan of the strains used in this study, monokaryotic ascospores were isolated from independent crosses of the respective strains (in the case of the *PaRcf1* deletion strain from a cross of wild type with Δ*PaRcf1*) and germinated for 3 days at 27 °C in the dark on BMM supplemented with 60 mM ammonium acetate. After germination, pieces of the resulting 3 day old mycelia were placed on M2 agar race tubes (M2 medium: 0.25 g/l KH_2_PO_4_, 0.3 g/l K_2_HPO_4_, 0.25 g/l MgSO_4_ × 7 H_2_O, 0.5 g/l urea and 10 g/l yellow dextrin. Addition of 2.5 μg/l biotin, 50 μg/l thiamine, 5 mg/l citric acid × 1 H_2_O, 5 mg/l ZnSO_4_ × 7 H_2_O, 1 mg/l Fe(NH_4_)_2_(SO_4_)_2_ × 6 H_2_O, 2.5 mg/l CuSO_4_ × 5 H_2_O, 25 μg/l MnSO_4_ × 1 H_2_O, 50 μg/l NaMoO_4_ × 2 H_2_O and 50 μg/l H_3_BO_4_ after sterilization of the basal medium) and incubated at 27 °C under constant light. The period of linear growth was recorded as lifespan in days. Growth rate was measured as growth of the mycelia in centimetres per day.

### Measurement of growth rate under stress conditions

To assess the susceptibility of the strains used in this study to paraquat-induced oxidative stress, monokaryotic ascospores were germinated as described above. After germination, pieces of the resulting 3 d old mycelia were placed on agar plates containing M2 medium supplemented with different concentrations of paraquat (0, 80, 160 or 320 μM) and incubated at 27 °C under constant light. Growth was recorded for 4 days and growth rate was expressed as growth of the mycelia in centimetres per day.

### Fertility analysis

Assessment of female fertility was essentially performed as described previously^48^. Isolates of wild type, Δ*PaRcf1* and Δ*PaRcf2*, in each case originating from monokaryotic ascospores, were grown on agar plates containing M2 medium at 27 °C under constant light for 13 days. Following spermatization at day 13, all plates were incubated for an additional three days at 27 °C, after which the total number of perithecia developing on each plate was counted. The mean number of perithecia developing per plate overgrown with the wild-type strain ‘s’ at 27 °C was defined as 1.

### Oxygen consumption measurement

To measure complex IV- and AOX-dependent oxygen consumption of wild type, Δ*PaRcf1* and Δ*PaRcf2*, monokaryotic ascospores of each strain were germinated as described above. After germination, pieces of the resulting 3 d old mycelia were grown for 2 days on M2 medium at 27 °C under constant light and subsequently under the same conditions in liquid CM for 3 days. Small pieces of mycelium (dry weight 2 to 10 mg) were then transferred into the ‘OROBOROS Oxygraph-2k’ (OROBOROS INSTRUMENTS) high-resolution respirometer and oxygen consumption was measured in liquid CM medium according to the manufacturer’s instructions. To inhibit respiration via complex IV, KCN was added to a final concentration of 1 mM. Respiration via AOX was inhibited by adding salicylhydroxamic acid (SHAM) to a final concentration of 4 mM. Absolute oxygen consumption was measured as pmol oxygen consumed per second and milligram dry weight mycelium. To express relative oxygen consumption after addition of specific respiratory inhibitors, absolute oxygen consumption of the respective strain (wild type, Δ*PaRcf1* or Δ*PaRcf2*) in the presence of KCN or SHAM was normalized to its total absolute oxygen consumption with no added inhibitors.

### Southern blot analysis

Total DNA of *P. anserina* was isolated with a well-established method for rapid extraction of nucleic acids from filamentous fungi^49^. DNA digestion, gel electrophoresis and Southern blotting were performed according to standard protocols. For Southern blot hybridization and detection, Digoxigenin-labeled hybridization probes (‘DIG DNA Labeling and Detection Kit’, Roche Applied Science) were used according to the manufacturer’s instructions.

The *PaRcf1*-specific hybridization probe was amplified by PCR using the oligonucleotides PaRcf1_A2 (5′-AGGAACCGCTCGTCCCAATC-3′) and PaRcf1_B2 (5′-CCTTGCCTGAGCAGCAACAC-3′) and corresponded to 371 nucleotides in exon 2 of *PaRcf1*. The *PaRcf2*-specific hybridization probe was amplified by PCR using the oligonucleotides PaRcf2_for1 (5′-AAGACGCCCACTTCAAGG-3′) and PaRcf2_rev2 (5′-TGGCTTCCGCTCAGATAC-3′) and corresponded to 408 nucleotides in exon 1 of *PaRcf2*. The *hph*-specific hybridization probe corresponded to the 727 bp ClaI-NcoI-fragment of the plasmid pKO7^44^. As a hybridization probe specific for the phleomycin resistance gene (*ble*), the 1293 bp BamHI-fragment of the plasmid pKO3^50^ was used.

### Western blot analysis

Mitochondrial protein extracts from *P. anserina* strains were isolated according to a previously developed procedure^25^ and further purified by discontinuous sucrose gradient (20-36–50%) ultracentrifugation^44^. Mitochondrial protein extracts for ‘OxyBlot’ analysis were isolated in the presence of 50 mM dithiothreitol and treated with the ‘OxyBlot™ Protein Oxydation Detection Kit’ (Merck Millipore) according to the manufacturer’s instructions. Separation of proteins by SDS-PAGE and subsequent transfer of proteins to PVDF membranes (Immobilon-FL, Millipore) were performed following standard protocols. Blocking and antibody incubation of blotted PVDF membranes were performed according to the Odyssey ‘Western Blot Analysis’ handbook (LI-COR).

Primary antibodies were raised against a PaCLPP (UniProt: B2B591) specific synthetic peptide ([Ac]-CGTMLSADAKEGKH-[OH]; NEP) corresponding to AA 242-254 (antibody dilution: 1:400), a PaLON (UniProt: B2AZ54) specific synthetic peptide ([H]-CDKIGRGYQGDPS-[OH]; Sigma) corresponding to AA 677-688 (antibody dilution: 1:1,500), a PaPRX (UniProt: B2AKR1) specific synthetic peptide ([Ac]-LHESSPGNKVNLADC-[NH2]; NEP) corresponding to AA 43-56 (antibody dilution: 1:2,000) and the PaPORIN (UniProt: B2B736) full-length protein (NEP; antibody dilution: 1:5,000). A human aconitase 2 antibody (abcam, product code: ab83528) used to detect *P*. *anserina* mitochondrial aconitase (UniProt: B2VLF5) was raised against a HsACO2 (UniProt: Q99798) specific recombinant fragment corresponding to AA 648-697 (antibody concentration: 1 μg/ml). A *Sauromatum guttatum* AOX antibody (Agrisera, product code: AS10 699) used to detect *P*. *anserina* AOX (UniProt: B2ACQ1) was raised against the SgAOX (UniProt: P22185) full-length protein (antibody dilution: 1:100). For detection of *P*. *anserina* HSP60 (UniProt: B2B270) an antibody (Stressgen, product code: SPA-807) raised against human full-length HSP60 (UniProt: P10809) was used (antibody concentration: 0.25 μg/ml). For detection of *P*. *anserina* SOD3 (UniProt: B2B5F1) an antibody (Stressgen, product code: SOD-111) raised against full-length rat SOD2 (UniProt: P07895) was used (antibody dilution: 1:2,000). Detection of PaRCF1 (UniProt: Q875C2) with a C-terminal 6xHis-tag was performed using a 6xHis-tag antibody (abcam, product code: ab9136; antibody dilution: 1:1,000). The *S*. *cerevisiae* COX2 (UniProt: P00410) antibody (antibody dilution: 1:5,000) used to detect PaCOX2 (UniProt: P20682) was a kind gift from Prof. T. Langer (Institute for Genetics, University of Cologne, Germany). Carbonyl groups of oxidized proteins derivatized to 2,4-dinitrophenylhydrazone (DNP-hydrazone) were detected with the DNP-specific antibody (antibody dilution: 1:100) provided with the ‘OxyBlot™ Protein Oxydation Detection Kit’ (Merck Millipore).

In all analyses, secondary antibodies conjugated with the infrared dyes IRDye 800CW or IRDye 680CW (LI-COR) were used (antibody dilution: 1:15,000–20,000). The ‘Odyssey Infrared Imaging System’ (LI-COR) was used for detection of western blots and densitometric quantification was performed with the image processing and analysis software ImageJ according to the developer’s documentation.

### BN-PAGE and complex IV ‘in-gel’ activity assay

BN-PAGE was performed according to the protocol described in detail by Wittig and colleagues^51^. For preparation of each sample, 100 μg of mitochondrial protein extracts were solubilized using a digitonin/protein ratio of 3:1 (w/w). Linear gradient gels (4–13%) overlaid with 3.5% stacking gels were used for separation of the solubilised samples. Respiratory chain components were then visualized by Coomassie blue staining and assigned as described previously^26^. To measure complex IV ‘in-gel’ activity, Coomassie blue staining was omitted and the gel was incubated in 50 mM phosphate buffer (pH 7.4) containing 1 mg/ml 3,3′-diaminobenzidine, 24 U/ml catalase, 1 mg/ml cytochrome c and 75 mg/ml sucrose^52^. Densitometric quantification was performed as described above.

### Statistical analysis

For statistical analysis of BN-PAGE, complex ‘in-gel’ activity, western blot and OxyBlot data as well as oxygen consumption measurements, two-tailed Student’s *t*-test was used. For statistical analysis of lifespan and growth rate, two-tailed Wilcoxon rank-sum test was used. If not explicitly stated otherwise, the respective samples were compared to the appropriate wild-type sample. *P*-values < 0.05 were considered statistically significant.

## Additional Information

**How to cite this article**: Fischer, F. *et al*. RCF1-dependent respiratory supercomplexes are integral for lifespan-maintenance in a fungal ageing model. *Sci. Rep*. **5**, 12697; doi: 10.1038/srep12697 (2015).

## Supplementary Material

Supplementary Information

## Figures and Tables

**Table 1:** Quantification of ETC complexes and supercomplexes in wild-type, Δ*PaRcf1* and Δ*PaRcf2* mitochondria.

(AU)	Wild type	Δ*PaRcf1*	Δ*PaRcf2*
**I_1_III_2_IV_2_ (S_2_)**	1.00 ± 0.20	0.07 ± 0.01	0.95 ± 0.33
**I_1_III_2_IV_1_ (S_1_)**	1.00 ± 0.08	0.03 ± 0.02	1.11 ± 0.18
**I_1_III_2_IV_0_ (S_0_)**	1.00 ± 0.38	15.30 ± 2.58	1.16 ± 0.16
**V_2_**	1.00 ± 0.13	1.00 ± 0.08	1.10 ± 0.08
**I_1_**	1.00 ± 0.29	1.33 ± 0.18	1.36 ± 0.18
**V_1_/III_2_**	1.00 ± 0.13	1.12 ± 0.12	1.15 ± 0.04
**IV_1_**	1.00 ± 0.43	–	1.43 ± 0.42
	(*n* = 4)	(*n* = 3)	(*n* = 4)

Quantitative BN-PAGE analysis of ETC complexes and supercomplexes in mitochondrial protein extracts from wild type (*n* = 4), Δ*PaRcf1* (*n* = 3) and Δ*PaRcf2* (*n* = 4). Densitometric quantification after Coomassie staining of BN gels was performed with the image processing and analysis software ‘ImageJ’ according to the developer’s documentation. Optical densities of the different complexes and supercomplexes were normalized to total Coomassie staining of the corresponding lane. The mean wild-type abundances were defined as 1. Data are mean protein abundance ± s.e.m. in arbitrary units (AU).

**Table 2:** Quantification of complex IV activity in wild-type, Δ*PaRcf1* and Δ*PaRcf2* mitochondria.

(%)	Wild type	Δ*PaRcf1*	Δ*PaRcf2*
**I_1_III_2_IV_2_ (S_2_)**	2.5 ± 0.2	0.5 ± 0.2	3.1 ± 0.9
**I_1_III_2_IV_1_ (S_1_)**	4.7 ± 0.5	1.3 ± 0.3	3.9 ± 0.6
**IV_1_**	92.8 ± 0.8	5.5 ± 1.4	92.9 ± 1.5
**IV_B_**	–	92.7 ± 1.8	–
	(*n* = 4)	(*n* = 4)	(*n* = 4)

Quantitative complex IV ‘in-gel’ activity assay with mitochondrial protein extracts from wild type (*n* = 4), Δ*PaRcf1* (*n* = 4) and Δ*PaRcf2* (*n* = 4). As a measure for the relative distribution of activities of monomeric complex IV and complex IV-containing supercomplexes within each individual strain, optical densities of bands representing complex IV activity were quantified and normalized to total complex IV activity in the corresponding lane. Data are mean complex IV activity ± s.e.m. in percentage.

**Table 3:** Lifespan and growth rate of wild type, Δ*PaRcf1*, Δ*PaRcf2*, Δ*PaRcf1*/*PaRcf1*-6xHis and *PaRcf1*-6xHis_Oex.

	Wild type	Δ*PaRcf1*	Δ*PaRcf2*	Δ*PaRcf1*/*PaRcf1*-6xHis	*PaRcf1*-6xHis_OEx
**Lifespan(d)**	24.5 ± 0.4	14.5 ± 0.7	24.2 ± 0.4	22.3 ± 0.5	27.6 ± 0.3
**Growth rate (cm d^−1^)**	0.59 ± 0.01	0.25 ± 0.01	0.64 ± 0.01	0.63 ± 0.01	0.65 ± 0.01
	(*n* = 98)	(*n* = 71)	(*n* = 31)	(*n* = 19)	(*n* = 26)

Lifespan and growth rate of wild type (*n* = 98), Δ*PaRcf1* (*n* = 71), Δ*PaRcf2* (*n* = 31), Δ*PaRcf1*/*PaRcf1*-6xHis (*n* = 19) and *PaRcf1*-6xHis_Oex (*n* = 26). Data are mean lifespan ± s.e.m. in days or mean growth rate ± s.e.m. in centimetres per day.
