# Supplementary material for: RCF1-dependent respiratory supercomplexes are integral for lifespan-maintenance in a fungal ageing model
Source: Sci Rep. 2015 Jul 29;5:12697. doi: 10.1038/srep12697 (PMC4518240; doi:10.1038/srep12697)
Supplement: Supplementary Information [file srep12697-s1.pdf]

RCF1-dependent respiratory supercomplexes are integral for lifespan-maintenance in a fungal ageing model

Fabian Fischer, Christodoulos Filippis & Heinz D. Osiewacz

SUPPLEMENTARY INFORMATION

Supplementary Figures S1 to S13

Supplementary Table S1

Supplementary Methods

Supplementary References

SUPPLEMENTARY FIGURES

a

```
PaRCF1             1 MSNGPLSNRPLPSSFDSNDDFYNENGF-QKVLRRLKEEPLVPIGCLLTVA     49
                     ||.       :|||||..:...::..| ::::...|::||||||||||..
ScRCF1             1 MSR-------MPSSFDVTERDLDDMTFGERIIYHCKKQPLVPIGCLLTTG     43

PaRCF1            50 AFTNAYRAMRRGDHAKVQKMFRARVAAQAFTVVAMVAGGMYY-------Q     92
                     |...|.:.:|.|:..|.|..||.||..||.|:||:|||...|       :
ScRCF1            44 AVILAAQNVRLGNKWKAQYYFRWRVGLQAATLVALVAGSFIYGTSGKELK     93

PaRCF1            93 ADRHKQKELWKLRQQKDAEEKHQKWIRELEARDAEEKALQERLDKRRKRA    142
                     |...:.||..|:|:        :.||:|||.|:.|.:|.::|.:..|.:.
ScRCF1            94 AKEEQLKEKAKMRE--------KLWIQELERREEETEARRKRAELARMKT    135

PaRCF1           143 AERAGGTGTESVAAQARAALRESKAGKTETGEATSTEANQADGGVLGSLG    192
                     .|..    .|....:...:..|:|.||.
ScRCF1           136 LENE----EEIKNLEKELSDLENKLGKK----------------------    159

PaRCF1           193 GWFGGSKKAPEDTTPALESKPEDPKN     218

ScRCF1           160 --------------------------     159
```

MTS, HIG_1_N, TMH

```
# Matrix: EBLOSUM62
# Length: 226
# Identity:      59/226 (26.1%)
# Similarity:    89/226 (39.4%)
# Gaps:          75/226 (33.2%)
# Score: 206.0
```

b

```
PaRCF2             1 MKIISKEEEDAHFKVVLKGGLIGGSVGLALGLGGVIAGSKRYPTIR--NL     48
                     |||::::|.:||....||||:.|...|.|:.........:|||..:  .|
ScRCF2             1 MKILTQDEIEAHRSHTLKGGIEGALAGFAISAIIFKVLPRRYPKFKPSTL     50

PaRCF2            49 TLPFRSFLVTSTGTFGAIVWAERYSIDFQRSHDSMYNYMDASHKAAAEAR     98
                     |...::.|..:..|....:.||..|.:|..   :||....:|..|..|.|
ScRCF2            51 TWSIKTALWITPPTVLTAICAEEASNNFDA---TMYGSGSSSEDALDEHR     97

PaRCF2            99 A-AAKSDTEKLMDWGRENRYSIVFTSWIAAMGLALAMVGKNKYLSGSQKL    147
                     . .:.|..:|.::....|:|.|:..:|.|::..:..:|.|:..::.:||:
ScRCF2            98 RWKSLSTKDKFVEGLSNNKYKIITGAWAASLYGSWVIVNKDPIMTKAQKI    147

PaRCF2           148 VQARMYAQGLTLAVLIATAAFETADAKAGKG--------RWETVMVVDPE    189
                     ||||||||.:|:.:|:|:......:.|....        |||..:.|
ScRCF2           148 VQARMYAQFITVGLLLASVGLSMYENKLHPNKQKVNEMRRWENALRV---    194

PaRCF2           190 DPEHKHLIEKRVRKEDYEGQNLWQDMVEAEERRLAEQKKHVAALEKKEKA    239
                      .|.:..:||..|:..|         |..|||..::..|.
ScRCF2           195 -AEEEERLEKEGRRTGY---------VSNEERINSKIFKS----------    224

PaRCF2           240 PAS     242

ScRCF2           225 ---     224
```

HIG_1_N, TMH

```
# Matrix: EBLOSUM62
# Length: 253
# Identity:      67/253 (26.5%)
# Similarity:   111/253 (43.9%)
# Gaps:          40/253 (15.8%)
# Score: 205.5
```

Supplementary Figure S1: Amino acid sequence alignments of PaRCF1 and PaRCF2 with their yeast homologues.

(**a**) Amino acid sequence alignment of *P. anserina* RCF1 (PaRCF1; UniProt: Q875C2) and *S. cerevisiae* RCF1 (ScRCF1; UniProt: Q03713) using EMBOSS Needle (http://www.ebi.ac.uk/Tools/psa/emboss_needle/). Lines indicate identical and dots similar amino acids. Predicted mitochondrial targeting sequences (MTS; http://ihg.gsf.de/ihg/mitoprot.html) are shown in green and ‘hypoxia induced protein conserved region’ domains (HIG_1_N; http://www.ncbi.nlm.nih.gov/Structure/cdd/wrpsb.cgi) in yellow. Predicted transmembrane helices (TMH; http://www.cbs.dtu.dk/services/TMHMM-2.0/) are underlined.

(**b**) Amino acid sequence alignment of *P. anserina* RCF2 (PaRCF2; UniProt: B2AAL7) and *S. cerevisiae* RCF2 (ScRCF2; UniProt: P53721) as in (a).

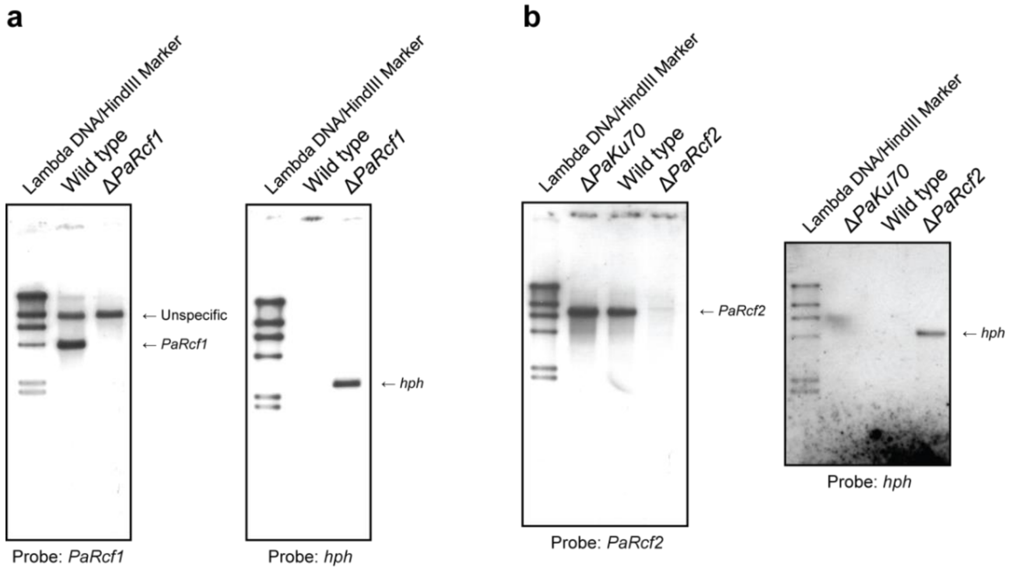

Supplementary Figure S2: Full Southern blots corresponding to Fig. 1b and c in the main paper. (**a**) Southern blot analysis of HindIII-digested genomic DNA (gDNA) from wild type and Δ*PaRcf1*. A *PaRcf1*-specific hybridization probe detects the 4217 bp *PaRcf1*-fragment only in wild-type gDNA. A 2659 bp fragment containing the hygromycin B phosphotransferase (*hph*) gene is detected only in gDNA of Δ*PaRcf1*. (**b**) Southern blot analysis of HindIII-digested gDNA from wild type and Δ*PaRcf2*. A *PaRcf2*-specific hybridization probe detects the 7513 bp *PaRcf2*-fragment only in wild-type gDNA. A 4667 bp fragment containing the hygromycin B phosphotransferase gene is detected only in gDNA of Δ*PaRcf2*.

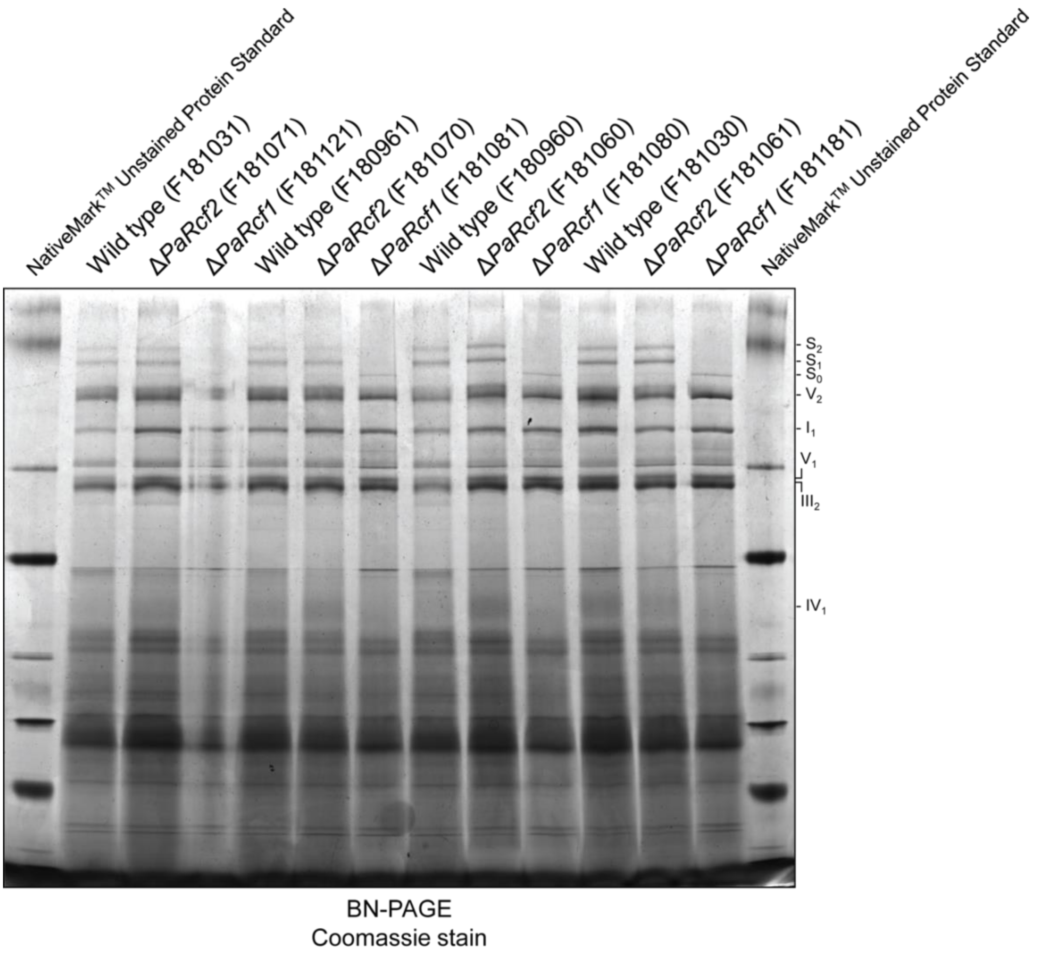

Supplementary Figure S3: Full Coomassie stained BN gel corresponding to Fig. 1d and Table 1 in the main paper. BN-PAGE analysis of mitochondrial protein extracts from wild type, Δ*PaRcf1* and Δ*PaRcf2*. The $I_1III_2IV_{0\text{-}2}$ ($S_{0\text{-}2}$) supercomplexes, dimeric complexes III and V (III_2 and V_2) as well as monomeric complexes I, IV and V were visualized by Coomassie staining. The strings in parentheses (e.g. F181031) denote specific monokaryotic isolates (i.e. biological replicates of the respective strains) from which the mitochondrial protein extracts were obtained.

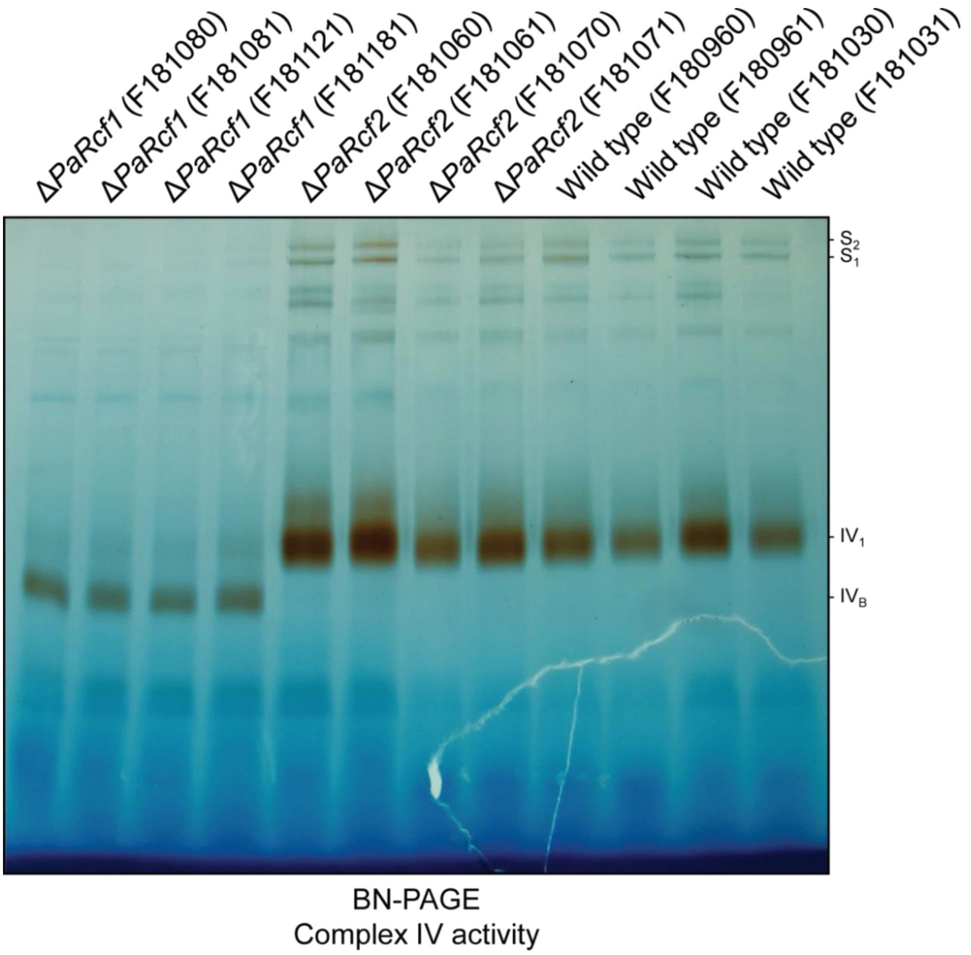

Supplementary Figure S4: Full BN gel with complex IV 'in-gel' activity staining corresponding to Fig. 1e and Table 2 in the main paper. Complex IV 'in-gel' activity assay with mitochondrial protein extracts from wild type, Δ*PaRcf1* and Δ*PaRcf2* to visualize activity of monomeric complex IV (IV_1 or IV_B) and complex IV-containing supercomplexes $I_1III_2IV_{1-2}$ (S_{1-2}). The strings in parentheses (e.g. F181080) denote specific monokaryotic isolates (i.e. biological replicates of the respective strains) from which the mitochondrial protein extracts were obtained.

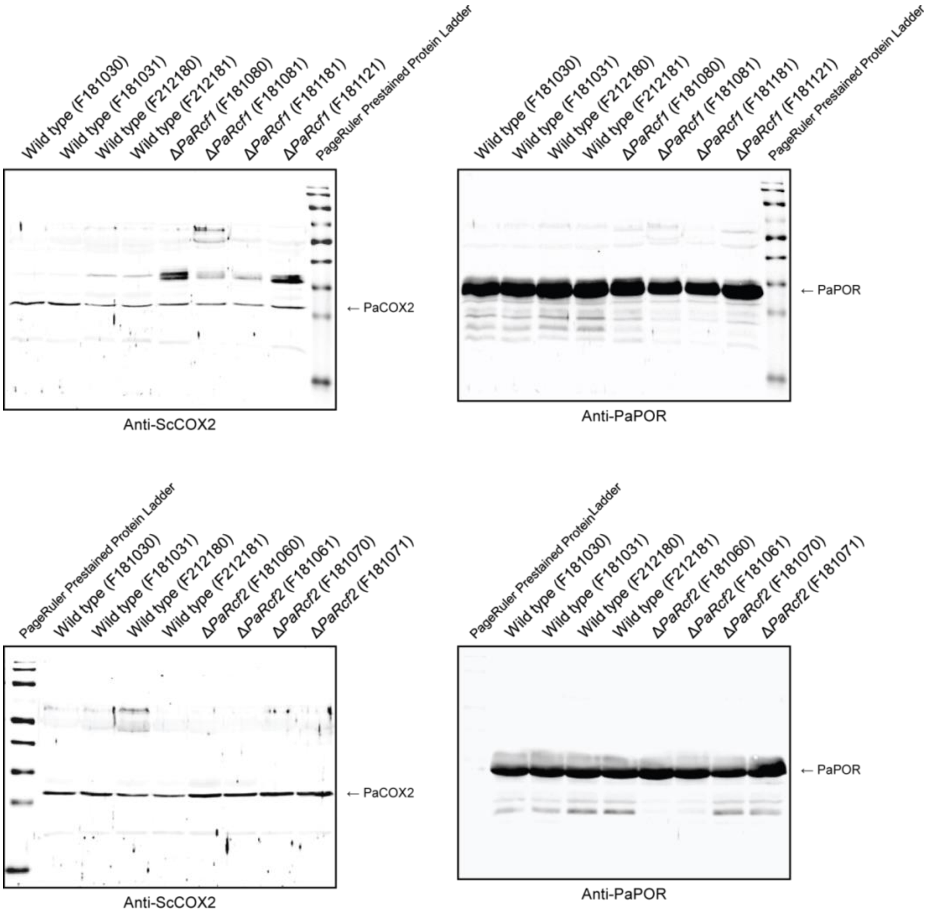

Supplementary Figure S5: Full western blots corresponding to Fig. 1f and g in the main paper. Western blot analyses of mitochondrial protein extracts from wild type and Δ*PaRcf1* (above) or wild type and Δ*PaRcf2* (below). A ScCOX2-specific antibody was used to detect the ~29 kDa PaCOX2 subunit of complex IV. PaPORIN (PaPOR) was detected as a loading control. The strings in parentheses (e.g. F181030) denote specific monokaryotic isolates (i.e. biological replicates of the respective strains) from which the mitochondrial protein extracts were obtained. Mitochondrial protein extract from the wild-type isolate F181030 served as a reference sample, allowing comparison of the two western blots.

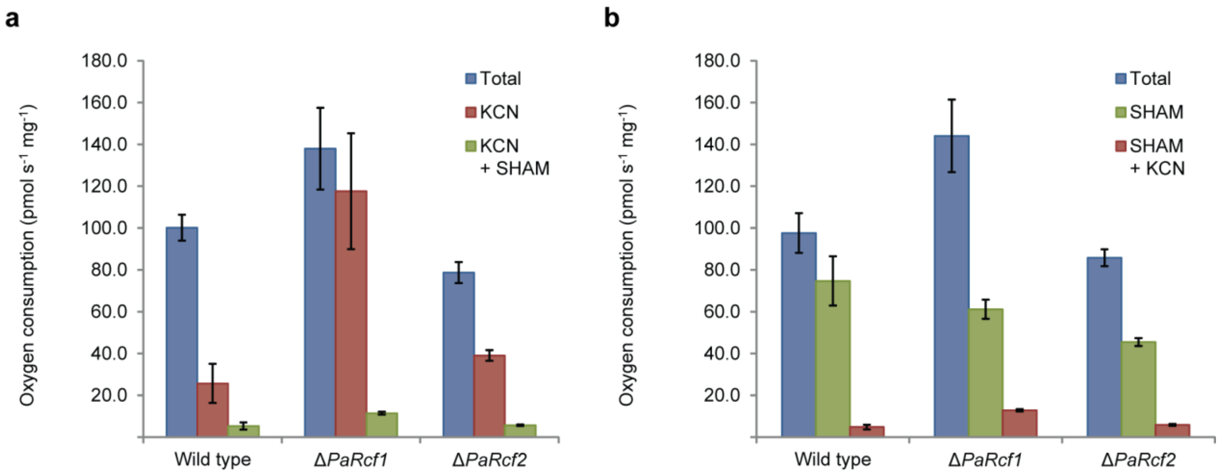

Supplementary Figure S6: Absolute oxygen consumption measurements corresponding to Fig. 2a in the main paper. (**a**) Oxygen consumption of wild-type (*n* = 3), Δ*PaRcf1* (*n* = 3) and Δ*PaRcf2* (*n* = 3) mycelium was measured before and after the sequential of complex IV- (KCN) and AOX-specific (SHAM) respiratory inhibitors. Data are mean absolute oxygen consumption ± s.e.m in pmol oxygen consumed per second and milligram mycelium. Total oxygen consumption (i.e. no added inhibitors) of wild type (100 ± 6 pmol s^{-1} mg^{-1}) is not significantly different from that of Δ*PaRcf1* (138 ± 20 pmol s^{-1} mg^{-1}; *P* = 1.9E-01) or Δ*PaRcf2* (79 ± 5 pmol s^{-1} mg^{-1}; *P* = 6.0E-02). Oxygen consumption of wild type after addition of KCN, compared to its total oxygen consumption, is significantly reduced (26 ± 9 pmol s^{-1} mg^{-1}; *P* = 4.4E-03). Additional inhibition of respiration with SHAM further reduces oxygen consumption of wild type (5 ± 2 pmol s^{-1} mg^{-1}; *P* = 1.6E-01). Oxygen consumption of Δ*PaRcf1* after addition of KCN, compared to its total oxygen consumption, is only slightly reduced (118 ± 28 pmol s^{-1} mg^{-1}; *P* = 4.2E-04). Additional inhibition of respiration with SHAM, however, strongly reduces oxygen consumption of Δ*PaRcf1* (12 ± 1 pmol s^{-1} mg^{-1}; *P* = 6.2E-02). Oxygen consumption of Δ*PaRcf2* after addition of KCN, compared to its total oxygen consumption, is significantly reduced (39 ± 3 pmol s^{-1} mg^{-1}; *P* = 6.1E-03) and further significantly reduced after additional inhibition of respiration with SHAM (6 ± 1 pmol s^{-1} mg^{-1}; *P* = 5.1E-03). *P*-values were determined in comparison to the respective sample by two-tailed Student's *t*-test. (**b**) Oxygen consumption of wild-type (*n* = 3), Δ*PaRcf1* (*n* = 3) and Δ*PaRcf2* (*n* = 3) mycelium was measured before and after the sequential addition of AOX- (SHAM) and complex IV-specific (KCN) respiratory inhibitors. Data are as in (a). Total oxygen consumption (i.e. no added inhibitors) of wild type (98 ± 9 pmol s^{-1} mg^{-1}) is not significantly different from that of Δ*PaRcf1* (144 ± 17 pmol s^{-1} mg^{-1}; *P* = 9.8E-02) or Δ*PaRcf2* (86 ± 4 pmol s^{-1} mg^{-1}; *P* = 3.4E-01). Oxygen consumption of wild type after addition of SHAM, compared to its total oxygen consumption, is only slightly reduced (75 ± 12 pmol s^{-1} mg^{-1}; *P* = 2.1E-01) but strongly and significantly reduced after additional inhibition of respiration with KCN (5 ± 1 pmol s^{-1} mg^{-1}; *P* = 2.6E-02). Oxygen consumption of Δ*PaRcf1* after addition of SHAM, compared to its total oxygen consumption, is significantly reduced (61 ± 5 pmol s^{-1} mg^{-1}; *P* = 3.4E-02) and further significantly reduced after additional inhibition of respiration with KCN (13 ± 1 pmol s^{-1} mg^{-1}; *P* = 8.3E-03). Oxygen consumption of Δ*PaRcf2* after addition of SHAM, compared to its total oxygen consumption, is significantly reduced (46 ± 2 pmol s^{-1} mg^{-1}; *P* = 3.5E-03) and further significantly reduced after additional inhibition of respiration with KCN (6 ± 1 pmol s^{-1} mg^{-1}; *P* = 1.6E-03). *P*-values were determined as in (a).

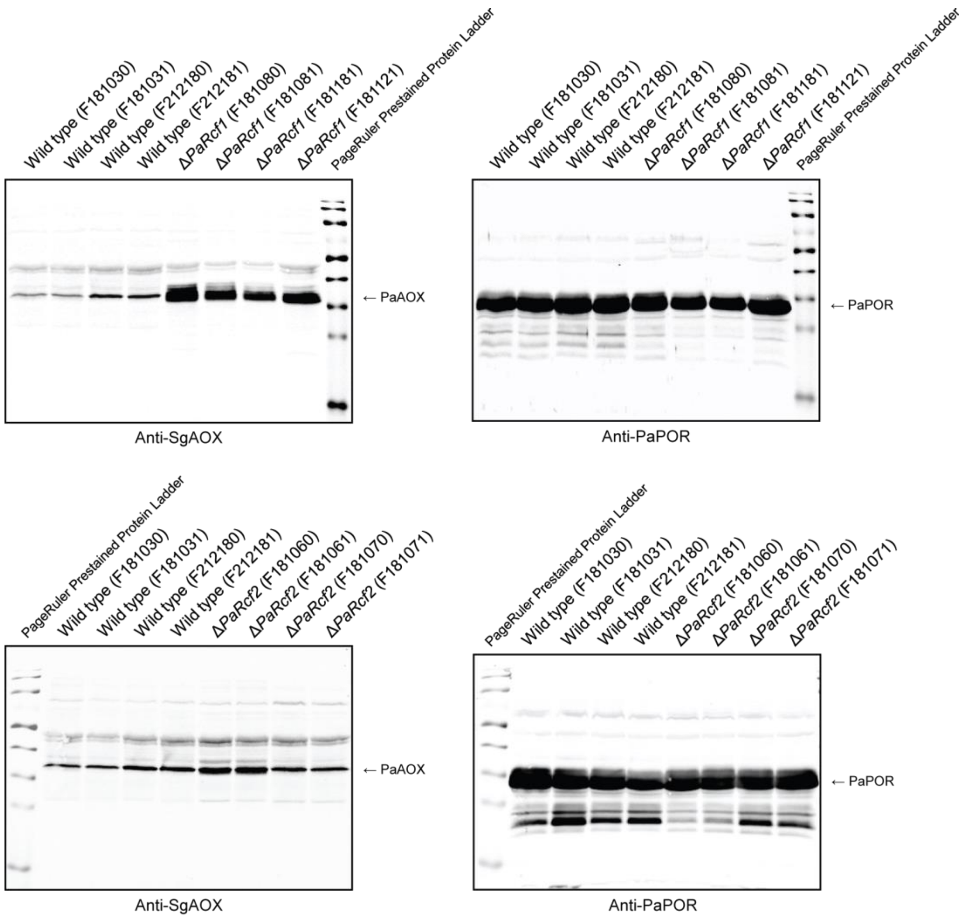

Supplementary Figure S7: Full western blots corresponding to Fig. 2b and c in the main paper. Western blot analyses of mitochondrial protein extracts from wild type and Δ*PaRcf1* (above) or wild type and Δ*PaRcf2* (below). A SgAOX-specific antibody was used to detect the ~34 kDa PaAOX. PaPORIN (PaPOR) was detected as a loading control. The strings in parentheses (e.g. F181030) denote specific monokaryotic isolates (i.e. biological replicates of the respective strains) from which the mitochondrial protein extracts were obtained. Mitochondrial protein extract from the wild-type isolate F181030 served as a reference sample, allowing comparison of the two western blots.

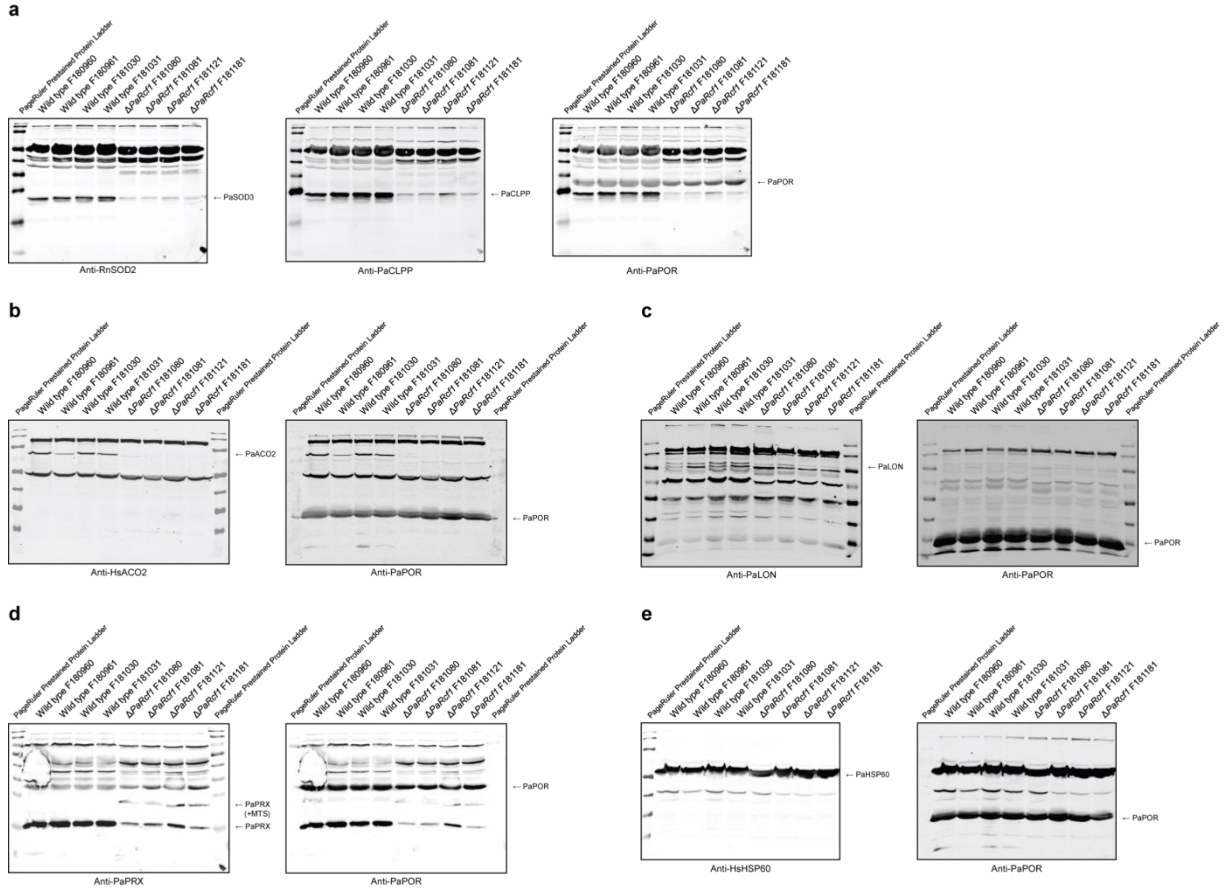

Supplementary Figure S8: Full western blots corresponding to Fig. 2g and h in the main paper. (**a**) Western blot analysis of mitochondrial protein extracts from wild type and Δ*PaRcf1*. A rat SOD2-specific antibody (Anti-RnSOD2) was used to detect the ~23 kDa mitochondrial PaSOD3. The ~25 kDa PaCLPP monomer was detected with a specific antibody. PaPORIN (PaPOR) was detected as a loading control. The strings in parentheses (e.g. F180961) denote specific monokaryotic isolates (i.e. biological replicates of the respective strains) from which the mitochondrial protein extracts were obtained. (**b**) Western blot analysis as in (a). A human mitochondrial aconitase-specific antibody (Anti-HsACO2) was used to detect the ~83 kDa PaACO2 tricarboxylic acid cycle enzyme. (**c**) Western blot analysis as in (a). The ~117 kDa PaLON mitochondrial matrix protease was detected with a specific antibody. (**d**) Western blot analysis as in (a). The ~17 kDa mitochondrial peroxiredoxin PaPRX was detected with a specific antibody. (**e**) Western blot analysis as in (a). A human HSP60-specific antibody (Anti-HsHSP60) was used to detect the ~59 kDa PaHSP60 mitochondrial chaperone.

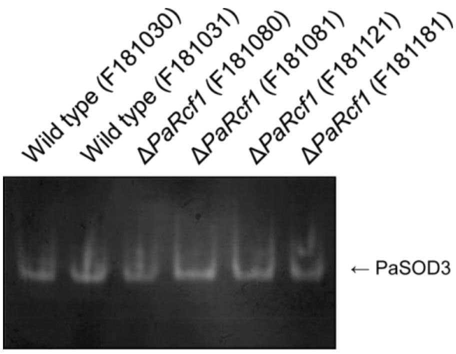

Supplementary Figure S9: Mitochondrial SOD activity in wild type and Δ*PaRcf1* appears identical. SOD ‘in-gel’ activity assay with mitochondrial protein extracts from wild type and Δ*PaRcf1* to visualize activity of the mitochondrial superoxide dismutase PaSOD3. The strings in parentheses (e.g. F181030) denote specific monokaryotic isolates (i.e. biological replicates of the respective strains) from which the mitochondrial protein extracts were obtained.

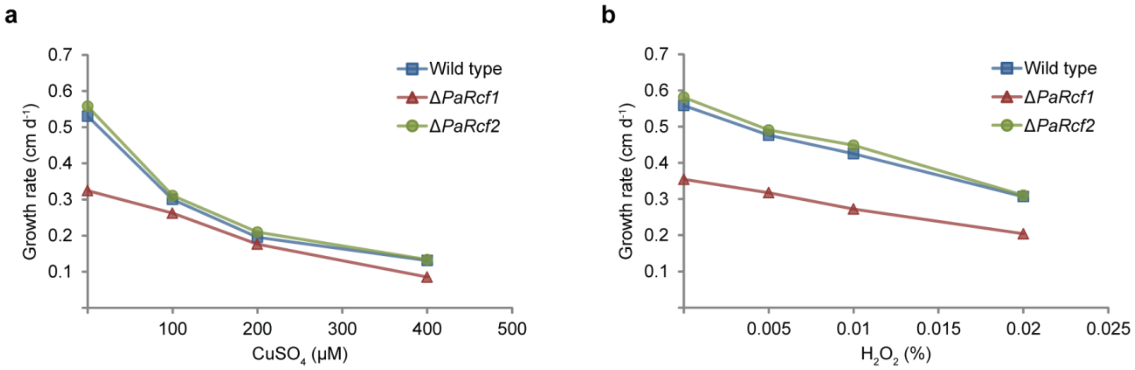

Supplementary Figure S10: Neither Δ*PaRcf1* nor Δ*PaRcf2* display increased susceptibility to $CuSO_4$ or H_2O_2. (**a**) Growth rate of wild type (n = 20), Δ*PaRcf1* (n = 20) and Δ*PaRcf2* (n = 20) on M2-medium with 0, 100, 200 or 400 µM of $CuSO_4$. Data are mean growth rate in centimetres per day. (**b**) Growth rate of wild type (n = 20), Δ*PaRcf1* (n = 20) and Δ*PaRcf2* (n = 20) on M2-medium with 0, 0.005, 0.01 or 0.02 % of H_2O_2. Data are mean growth rate in centimetres per day.

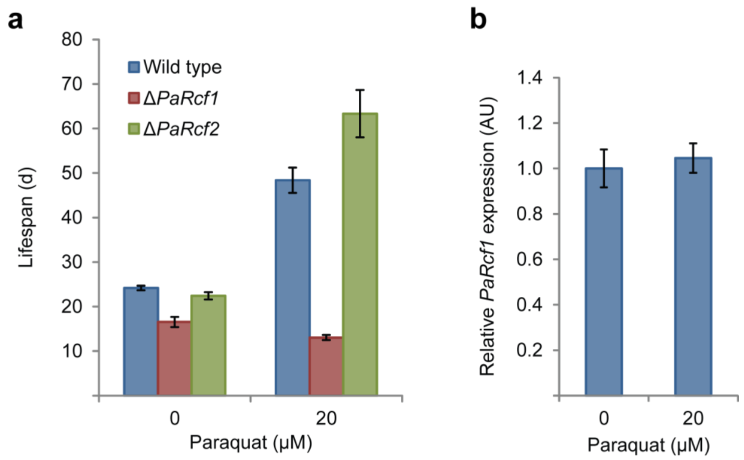

Supplementary Figure S11: Paraquat-triggered lifespan extension is abrogated in Δ*PaRcf1*, but *PaRcf1* expression is not induced by paraquat. **(a)** Lifespan of wild type (*n* = 24), Δ*PaRcf1* (*n* = 24) and Δ*PaRcf2* (*n* = 12) on M2-medium without (wild type: 24.7 ± 0.5 | Δ*PaRcf1*: 16.5 ± 1.2; *P* = 1.4E-05 | Δ*PaRcf2*: 22.4 ± 0.8; *P* = 6.3E-02) or with (wild type: 48.4 ± 2.9 | Δ*PaRcf1*: 13.0 ± 0.6; *P* = 2.9E-09 | Δ*PaRcf2*: 63.3 ± 5.3; *P* = 1.6E-02) 20 µM paraquat. Data given in parentheses are mean lifespan ± s.e.m. in days. *P*-values were determined in comparison to the wild-type sample by two-tailed Wilcoxon rank-sum test. (**b**) Relative *PaRcf1* expression in wild type (*n* = 8) cultivated without (1.00 ± 0.08) or with (1.05 ± 0.06; *P* = 0.68 by two-tailed Student's *t*-test) 20 µM paraquat as determined by RT-qPCR. *PaRcf1* expression was normalized to that of the reference gene *PaPorin* and the mean expression in wild type cultivated without paraquat was defined as 1. Data given in parentheses are mean *PaRcf1* expression ± s.e.m. in arbitrary units (AU).

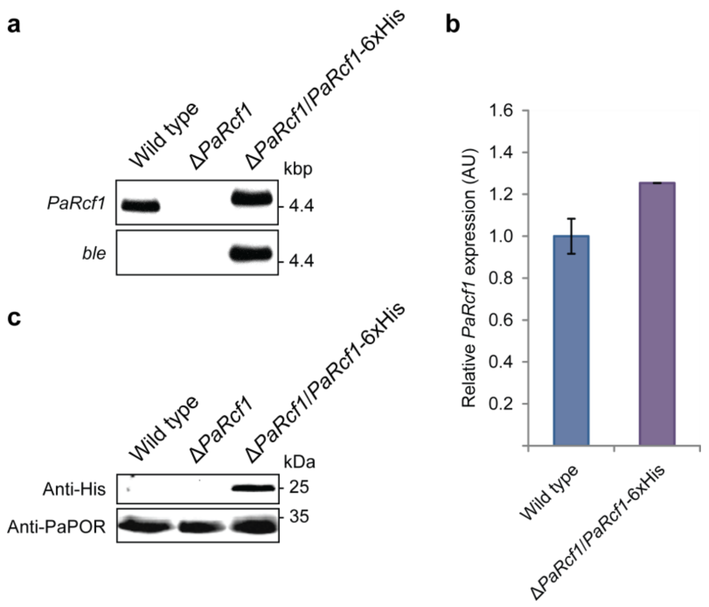

Supplementary Figure S12: Generation and verification of the complemented *PaRcf1* deletion strain Δ*PaRcf1*/*PaRcf1*-6xHis. (**a**) Southern blot analysis of HindIII-digested genomic DNA (gDNA) from wild type, Δ*PaRcf1* and Δ*PaRcf1*/*PaRcf1*-6xHis. A *PaRcf1*-specific hybridization probe was used for detection of the wild-type and recombinant gene fragment. The phleomycin resistance gene (*ble*) is detected by a *ble*-specific hybridization probe. (**b**) Relative *PaRcf1* expression in wild type (1.00 ± 0.08; n = 8) and Δ*PaRcf1*/*PaRcf1*-6xHis (1.25 ± 0.01; n = 2; P = 1.9E-02 by two-tailed Student's t-test) as determined by RT-qPCR. *PaRcf1* expression was normalized to that of the reference gene *PaPorin* and the mean expression in wild type was defined as 1. Data given in parentheses are mean *PaRcf1* expression ± s.e.m. in arbitrary units (AU). (**c**) Representative western blot analysis of mitochondrial protein extracts from wild type, Δ*PaRcf1* and Δ*PaRcf1*/*PaRcf1*-6xHis. A 6xHis-specific antibody was used to detect the ~23 kDa recombinant PaRCF1-6xHis protein. PaPORIN (PaPOR) was detected as a loading control.

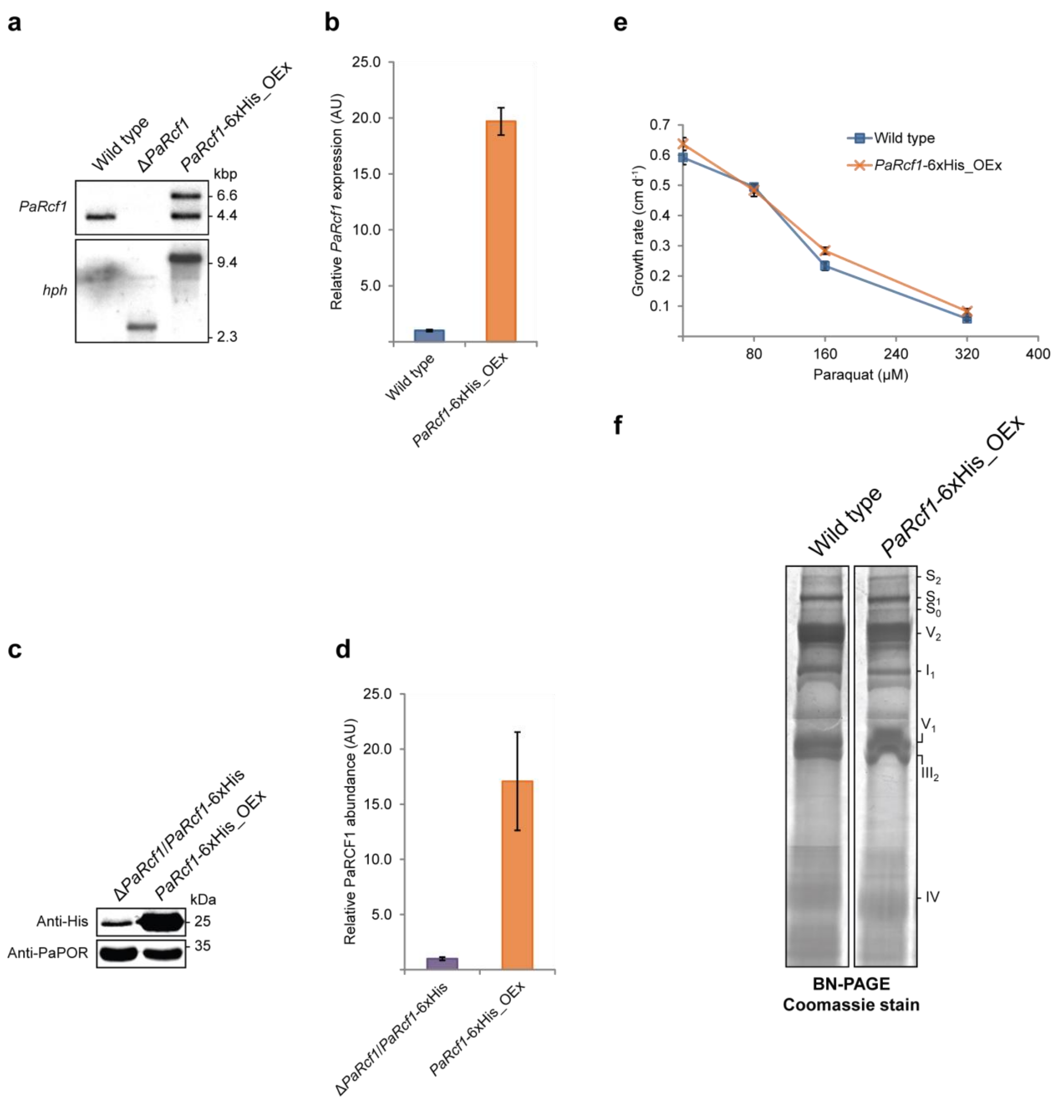

Supplementary Figure S13: Stable overexpression of *PaRcf1*-6xHis is possible but does not induce overt alterations in ETC organization. (**a**) Southern blot analysis of HindIII-digested genomic DNA (gDNA) from wild type, Δ*PaRcf1* and *PaRcf1*-6xHis_OEx. A *PaRcf1*-specific hybridization probe was used for detection of the wild-type and recombinant gene fragment. The hygromycin B phosphotransferase (*hph*) gene is detected by an *hph*-specific hybridization probe. (**b**) Relative *PaRcf1* expression in wild type (1.00 ± 0.08; n = 8) and *PaRcf1*-6xHis_OEx (19.70 ± 1.22; n = 3; P = 4.1E-03 by two-tailed Student's t-test) as determined by RT-qPCR. *PaRcf1* expression was normalized to that of the reference gene *PaPorin* and the mean expression in wild type was defined as 1. Data given in parentheses are mean *PaRcf1* expression ± s.e.m. in arbitrary units (AU). (**c**) Representative western blot analysis of mitochondrial protein extracts from Δ*PaRcf1*/*PaRcf1*-6xHis and *PaRcf1*-6xHis_OEx. A 6xHis-specific antibody was used to detect the ~23 kDa recombinant PaRCF1-6xHis protein. PaPORIN (PaPOR) was detected as a loading control. (**d**) Quantitative western blot analysis of mitochondrial protein extracts from Δ*PaRcf1*/*PaRcf1*-6xHis (1.00 ± 0.14; n = 3) and *PaRcf1*-6xHis_OEx (17.08 ± 4.45; n = 3; P = 3.6E-02 by two-tailed Student's t-test). The PaRCF1-6xHis abundance was normalized to that of PaPOR and the mean wild-type abundance was defined as 1. Data given in parentheses are mean PaRCF1-6xHis abundance ± s.e.m. in AU. (**e**) Growth rate of wild type (n = 15) and *PaRcf1*-6xHis_OEx (n = 15) on M2-medium with 0, 80, 160 or 320 µM of paraquat. Data are mean growth rate ± s.e.m. in centimetres per day. (**f**) Representative BN-PAGE analysis of mitochondrial protein extracts from wild type and *PaRcf1*-6xHis_OEx. The $I_1III_2IV_{0\text{-}2}$ ($S_{0\text{-}2}$) supercomplexes, dimeric complexes III and V (III_2 and V_2) as well as monomeric complexes I, IV and V were visualized by Coomassie staining.

SUPPLEMENTARY TABLES

Supplementary Table S1: Quantification of ETC complexes and supercomplexes in wild type and *PaRcf1*-6xHis_OEx mitochondria

(AU)	Wild type	*PaRcf1*-6xHis_Oex
$I_1III_2IV_2$ (S_2)	1.00 ± 0.20	0.82 ± 0.17
$I_1III_2IV_1$ (S_1)	1.00 ± 0.14	0.94 ± 0.08
$I_1III_2IV_0$ (S_0)	1.00 ± 0.64	0.50 ± 0.17
V_2	1.00 ± 0.04	1.08 ± 0.21
I_1	1.00 ± 0.14	0.92 ± 0.21
V_1/III_2	1.00 ± 0.12	0.95 ± 0.15
IV_1	1.00 ± 0.22	1.30 ± 0.22
	(*n* = 3)	(*n* = 3)

Quantitative BN-PAGE analysis of ETC complexes and supercomplexes in mitochondrial protein extracts from wild type (*n* = 3) and *PaRcf1*-6xHis_OEx (*n* = 3). Densitometric quantification after Coomassie staining of BN gels was performed with the image processing and analysis software ImageJ according to the developer's documentation. Optical densities of the different complexes and supercomplexes were normalized to total Coomassie staining of the corresponding lane. The mean wild-type abundances were defined as 1. Data are mean protein abundance ± s.e.m. in arbitrary units (AU).

SUPPLEMENTARY METHORDS

Measurement of growth rate under stress conditions

To assess the susceptibility of the strains used in this study to $CuSO_4$ and H_2O_2-induced oxidative stress, monokaryotic ascospores were germinated for 3 days at 27 °C in the dark on BMM supplemented with 60 mM ammonium acetate. After germination, pieces of the resulting 3 d old mycelia were placed on agar plates containing M2 medium supplemented with different concentrations of $CuSO_4$ (0, 100, 200 or 400 µM) or H_2O_2 (0, 0.005, 0.01 or 0.02 %) and incubated at 27 °C under constant light. Growth was recorded for 4 days and growth rate was expressed as growth of the mycelia in centimetres per day.

SOD 'in-gel' activity assay

For each sample, 100 µg of mitochondrial protein extracts were prepared with non-denaturing sample buffer and separated on native polyacrylamide gels (8.5 % separating gels overlaid with 5 % stacking gels). Staining of gels with nitroblue tetrazolium, riboflavin and tetramethylethylenediamine to visualize 'in-gel' SOD activity was performed according to the method described by Flohé and Ötting[1].

Reverse transcription quantitative real-time PCR (RT-qPCR)

To isolate total RNA of wild type, Δ*PaRcf1*/*PaRcf1*-6xHis and *PaRcf1*-6xHis_OEx, monokaryotic ascospores were isolated from independent crosses of the respective strains and germinated for 2 days at 27 °C in the dark on BMM supplemented with 60 mM ammonium acetate. After germination, pieces of the resulting 2 day old mycelia were placed on agar plates containing M2 medium with or without 20 µM paraquat and incubated for 3 days at 27 °C under constant light. The mycelium was then pulverized with liquid nitrogen and used for RNA isolation with the 'NucleoSpin® RNA Plant' kit (Macherey-Nagel) following the standard protocol, including an rDNase digest in solution. Reverse transcription of 1 µg RNA per sample was performed with the 'RevertAid Reverse Transcriptase' (Thermo Fisher Scientific). The cDNA was diluted to a concentration of 10 ng μl^{-1} and 20 ng were used for each qPCR reaction using the 'iQ™ SYBR® Green Supermix' (Bio-Rad) and the 'Myiq™' real-time PCR detection system (Bio-Rad) according to the manufacturer's instructions. Three technical replicates were performed for each sample. To amplify the *PaRcf1* transcript the oligonucleotides PaRcf1-qRT_for (5'-ACCCCTTCCCTCGTCATTTG-3') and PaRcf1-qRT_rev (5'-AGCTCCCTGATCCACTTCTG-3') were used. For amplification of the *PaPorin* transcript used for normalization the oligonucleotides PaPorin-qRT_for (5'-TCTCCTCCGGCAGCCTTG-3') and PaPorin-qRT_rev (5'-GAGGGTGTCGGCAAGTTC-3') were used. The real-time PCR efficiency for each transcript was calculated as described in detail by Pfaffl[2]. Relative *PaRcf1* expression was normalized to the expression of the reference gene *PaPorin* using the following formula:

$$\text{Relative expression} = \frac{{E_{PaPorin}}^{\mathrm{CP}(PaPorin)}}{{E_{PaRcf1}}^{\mathrm{CP}(PaRcf1)}}$$

E: Real time PCR efficiency and CP: Crossing point

SUPPLEMENTARY REFERENCES

[1] Flohé, L. & Ötting, F. Superoxide dismutase assays. *Methods Enzymol.* **105**, 93-104 (1984).

[2] Pfaffl, M. W. A new mathematical model for relative quantification in real-time RT-PCR. *Nucleic Acids Res.* **29**, e45 (2001).
